# Multi-Year Biofilm Formation on Granitic Surfaces Reveals Dynamic Microbial Communities in Fennoscandian Shield Deep Groundwaters

**DOI:** 10.1007/s00248-026-02812-4

**Published:** 2026-06-19

**Authors:** Magnus Ståhle, Anders Johnson, Stephanie Turner, Per Mårtensson, Birgitta Kalinowski, Mark Dopson

**Affiliations:** 1 Centre for Ecology and Evolution in Microbial Model Systems (EEMiS), Linnaeus University, Kalmar, Sweden; 2 Centre for the Environment (CENWIN), Linnaeus University, Kalmar, Sweden; 3 Swedish Nuclear Fuel and Waste Management Company, Solna, Stockholm, Sweden

**Keywords:** 16S rRNA gene, Äspö hard rock laboratory, Carbon fixation, Nitrogen fixation, Sulfur cycling

## Abstract

The deep terrestrial biosphere is the vast biome beneath the soil layer that contains the majority of the Earth’s prokaryotic biomass, yet it is one of the least investigated communities. Although, estimates of deep biosphere biomass suggest biofilm cells outnumber the planktonic biomass by several orders of magnitude, most investigations target planktonic communities captured from groundwaters. This multi-year study used 16S rRNA gene sequencing to compare planktonic and biofilm communities attached to natural granitic rock, demonstrating that biofilm formation selected for taxa with distinct relative abundances and exhibited temporal development. The biofilm communities also showed a decreasing influence of introduced populations on the natural rock surfaces (macadam) present at the onset of the incubations. After two- and four-years of biofilm incubation, a community developed that was dominated by sulfur/sulfate reducing Desulfocapsaceae, Desulfobacteraceae, and BM004 along with the families UBA5619, Rhodocyclaceae, Profunditerraquicolaceae, and UBA2206. This long-term community included populations predicted to be host-associated ultra-small cells. This contrasted with previous studies of early biofilm development in deep Fennoscandian Shield groundwaters that suggested biofilm initiation was mediated by lithotrophic carbon and nitrogen fixing populations. However, metabolic predictions based upon the 16S rRNA gene-based communities also showed an autotrophic and diazotrophic community including sulfur cycling in line with the previous studies. In conclusion, this study showed long-term biofilm composition to be dissimilar to the planktonic communities with a consistent strategy for energy conservation similar to previous studies of early biofilm formation from these groundwaters.

**Supplementary Information:**

The online version contains supplementary material available at 10.1007/s00248-026-02812-4.

## Introduction

The deep terrestrial biosphere encompasses the life below the soil horizon that exists in water filled bedrock fractures that was first postulated by Gold [1]. The deep biosphere extends to approximately 5 km in depth [2] and is constrained by temperature with most of the Earth’s crust being above approximately 122 °C for the majority of time and thus, uninhabitable [3]. The deep terrestrial biosphere is one of the largest biomes on Earth and is estimated to constitute 90% of prokaryotes [4] and between 10 to 20% of all the planet’s biomass [2]. Despite the predominantly extremely low availability of carbon and energy, the deep biosphere contains active life from all three domains along with viroid particles (reviewed in [5, 6]). However, due to the vast size of the deep biosphere, it is one of the least understood environments that contains many novel microbial populations [7].

Microbial adaptations to the deep biosphere include ultra-small cells [8] and streamlined genomes [9]. These populations include the Patescibacteria and DPANN archaea that are often dominant in groundwaters [10, 11] and exhibit minimal biosynthetic capacities such that they are often reliant on (epi)symbioses with other prokaryotes [12]. The predominance of Patescibacteria in groundwaters has been attributed to their mobilization from the soil such that they can constitute up to 40% of the exported cells [13]. This ultra-slow-growing deep life is termed ‘aeonophilic’ [14] and is hypothesized to have an ‘episodic lifestyle’ in response to pulses of available energy [15]. Sub-surface life is important in global biogeochemical processes as well as the built environment such as for repositories of radioactive waste [16] and for hydrogen storage [17].

Biofilms comprise attached cells with both organization and structure [18–20] and are the dominant form of microbial life in all major habitats, except the open ocean. Estimates of deep terrestrial attached versus planktonic populations vary, but all suggest a large predominance of attached biomass [2, 20]. These biofilms may alter the hydrological regime. For example, bacteria indigenous to deep subsurface granitic groundwaters produced filamentous networks encrusted with aluminosilicates in a laboratory experiment designed to mimic in situ conditions, resulting in blocked pore spaces [21]. Conversely, the lithology of the rock surface can influence deep biosphere biofilm communities by providing access to electron donors that fuel chemolithotrophic metabolisms [22], selecting for populations capable of performing extracellular electron transfer [23], and providing a source of e.g., phosphorus from apatite [24]. Furthermore, the microbial populations associated with different weathered crystals and whole-rock granite were driven by the mineral chemical composition [25]. Equally, microorganisms weather the rock surface and alter its structure such as by the formation of secondary minerals. These interactions result in the attached and planktonic microbial community compositions being largely distinct. For instance, 16S rRNA gene sequences most similar to a Betaproteobacteria comprise 54% of a sedimentary rock groundwater planktonic cells compared to 77% of the biofilm sequences being related to *Methylobacillus flagellatus* [26]. In addition, sessile fungal communities on mica schist in the Outokumpu Deep Drill Hole, Finland, also differed from the planktonic populations [27]. However, despite biofilm communities being quantitatively more relevant, most studies focus on planktonic populations, and little is known about biofilm communities and their development.

One site for deep biosphere research is the underground Äspö Hard Rock laboratory (HRL) that is a 3.6 km long tunnel reaching 460 m below sea level (mbsl) in 1.8 Ga old Proterozoic Fennoscandian Shield crystalline bedrock (also termed the Baltic Shield). Boreholes extend from the tunnel that intersect groundwater bearing fractures of varying age and geochemistry [28]. 16S rRNA gene-based surveys of Äspö HRL planktonic populations reveal that the majority of the cells are viable [29] and that the communities are shaped by the depth from the surface and organic carbon content in shallower populations but not the deepest old saline groundwaters [30]. An investigation of biofilm formation on garnet and glass surfaces in ‘modern marine’ and ‘old saline’ groundwaters shows initial biofilm formation was mediated by autotrophic and diazotrophic populations driven by hydrogen as electron donor [31]. A second flow-cell study of biofilm formation in these groundwaters identified a chemolithotrophic *Thiobacillus* population as a key member of the early biofilm forming community with RNA transcripts encoding inorganic sulfur oxidation as an electron donor along with biofilm related functions such as cell motility, quorum sensing, and extracellular matrix generation [32]. However, biofilm formation has not been investigated in the Äspö HRL with the local Fennoscandian Shield rock as the solid matrix for cell attachment. Furthermore, many studies focus on short-term biofilm formation, but long-term studies are more relevant for understanding biofilms in these slow-growing aeonophilic communities.

This study investigated long-term biofilm formation on natural rock from the Äspö HRL as compared to the planktonic community using 16S rRNA gene amplicon sequencing. The hypothesis tested was that biofilm formation was initiated by a subset of the total planktonic deep groundwater community and that cells with streamlined genomes will have low relative abundance in the initial biofilms. The study has relevance to both natural processes by furthering our understanding of the poorly characterized deep biosphere as well as anthropogenic settings.

## Materials and Methods

### Study Site and Microbial Groundwater Sampling

**Fig. 1:**
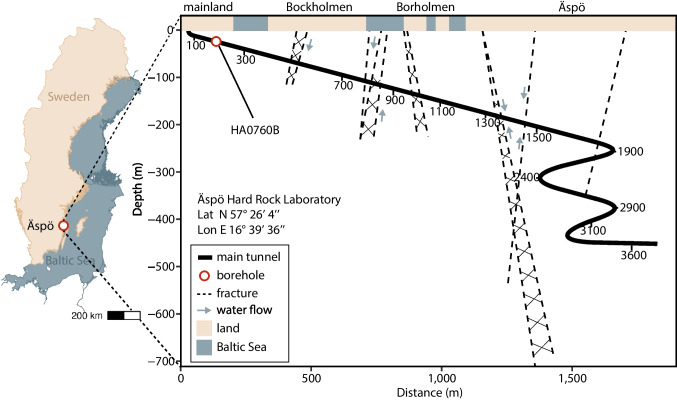
Map of Sweden showing the Äspö HRL location with the approximate position of the HA0760B borehole in the tunnel. The figure was modified from Lopez-Fernandez et al. [32] and Pedersen et al. [62]

The study was performed between 1 December 2020 and 23 January 2025 at the Swedish Nuclear Fuel and Waste Management Company (SKB) built Äspö HRL in southeastern Sweden (Lat N 57° 26′ 4″, Lon E 16° 39′ 36″; Fig. 1 & Supplemental Fig. 1). A flow diagram with a detailed explanation in the legend for all microbiology samples taken from groundwater, macadam that refers to the rocks from the Äspö HRL tunnel used for solid support prior to filling the containers, biofilm on the macadam surface, and planktonic cells flowing out of the four containers analyzed in the study is provided in Supplemental Fig. 2. The study design was such that (i) geochemical sampling was performed prior to the study along with multiple time points during the four-year study; (ii) the indigenous microbial community in the groundwater without passing through the containers with macadam was investigated before and after study; (iii) the macadam used as a solid support for biofilm formation was sampled each time before it was introduced into the containers; and (iv) the planktonic community from the container outlet that had passed through the macadam plus (v) the attached biofilm community on the macadam were captured by opening the containers and emptying the macadam after one-, two-, and four-years incubation to capture differences between the total planktonic population and the temporal development of the biofilm community.

Groundwater intersected by borehole HA0760B at 171.3 mbsl in the Äspö HRL tunnel was sampled for cell capture according to published methods [7, 32]. Briefly, stagnant water in the borehole was removed by flushing three section volumes (i.e., 3 × 72 L) before the sampling tubing and the high-pressure filter holders were flushed. Sterile 0.1 µm pore size filters were placed in the holders and left in place for the required water volume to pass. The filters were then aseptically collected, placed in sterile cryogenic tubes, immediately flash frozen in liquid nitrogen, and moved to a −80 °C freezer upon returning to Linnaeus University. The total volume of water that passed through the filters along with additional sample details are given in Supplemental Table 1. Biofilm was sampled by opening the containers and emptying them while immediately aseptically collecting macadam into sterile Falcon tubes from various depths within the containers to obtain a representative sample of the whole container biofilm. The samples were immediately flash frozen and stored at −80° C freezer upon returning to Linnaeus University. Contamination risks associated with the extremely low biomass in the deep biosphere are mitigated by the Äspö HRL being constructed approximately 30 years ago with the groundwaters flowing towards the tunnel by hydrostatic pressure.

### Containers for Biofilm Formation

Four containers with an internal volume of 100 L (Supplemental Fig. 1) were constructed, of which two were lined with Teflon as a retardant to microbial growth (Tef1 and Tef2) and two were unlined such that putative hydrogen generated by groundwater contact with the stainless-steel served as a microbial electron donor (SS1 and SS2). The containers were equipped with downstream needle valves to maintain the pressure within the system close to that in the borehole. A splitter with four valves (number of replicates (*n*) = 4) was used to divert groundwater flow through all four containers at a rate of 15–20 mL/min. The containers were filled with natural, non-sterilized ‘macadam’ of crystalline bedrock rock from the Äspö HRL crushed to a size of 16 to 32 mm to provide a solid surface for biofilm growth that was representative of fracture conditions. The macadam was added to all four containers at the start of the experiment on 10 December 2020 and to the SS1 and Tef1 containers on two occasions (14 December 2021 and 6 December 2022) after they had been emptied for sampling of the one-year incubation biofilms (see Supplemental Fig. 2 legend for details).

### Geochemistry Analyses

Groundwater samples for geochemistry (sampling times provided in Supplemental Table 2) were collected and analyzed according to established SKB groundwater monitoring protocols [33]. The water was analyzed for pH, conductivity, and carbonate alkalinity. Anions were analyzed with ion chromatography (DIN EN ISO 10 304–1: 2009) and DOC/TOC (dissolved organic carbon/total organic carbon) was analyzed using a Shimadzu Total Organic Carbon Analyzer (TOC-L CPH) with a total nitrogen unit analyzer TNM. Analysis of the water ^18^O/^16^O and ^2^H/^1^H ratio (per mil deviation from Standard Mean Ocean Water, SMOW) were determined by laser spectrometry (Los Gatos Research; Triple-Liquid Water Isotope Analyzer). Analyses for major ions were done by ALS Scandinavia using Inductively Coupled Plasma Atomic Emission Spectroscopy (ICP-AES).

### DNA Extraction, PCR Amplification, and 16S rRNA Gene Amplicon Sequencing Variants

Genomic DNA from groundwater planktonic cells captured on the 0.1 µm filters was extracted using the DNeasy PowerWater Kit (Qiagen). To test extraction methods for the biofilm cells on the macadam, either 15 mL lysis buffer from the DNeasy PowerSoil Kit or 15 mL 1 × phosphate buffered saline (PBS) were added to the 50 mL Falcon tubes and DNA extracted using the DNeasy PowerSoil Kit according to the kit protocol. As the two methods gave similar results, a single method was used for the remaining biofilm samples with the addition of 10 mL lysis buffer, vortexing at maximum speed for 10 min, decanting the liquid, larger particles were allowed to sediment for 3 min, and the liquid phase added directly to the DNeasy PowerWater Kit.

Partial 16S rRNA genes were amplified with a modified PCR protocol [34] using primers 341 F and 805R [35]. The PCR primers used in this study were designed to amplify bacteria although some archaeal lineages are amplified from Äspö HRL groundwaters [29, 30]. Illumina libraries were constructed from the PCR amplification products and sequenced at Science for Life Laboratory, Sweden (www.scilifelab.se).

### Bioinformatic Analyses

The 16S rRNA gene sequencing generated a total of 18,093.9 kreads (min: 0.26, max: 484.95 kreads per sample) that were processed to give between 0 and 1,954 amplicon sequencing variants (ASVs) per sample (Supplemental Table 1). Rarefaction curves of the number of ASVs versus sequencing reads showed asymptotic curves suggesting sufficient sequencing depth was achieved to identify most of the microbial diversity (Supplemental Fig. 3). All biofilm and new macadam samples were collected in at least triplicates. However, where fewer biological replicates were reported, it was due to insufficient DNA in the extraction for PCR amplification and 16S rRNA gene amplicon sequencing.

Raw sequencing reads were processed using the Nextflow (v25.04.7) Ampliseq pipeline (v2.15.0 [36]) that included FastQC (v0.12.1), Cutadapt (v4.6), MultiQC (v1.29), DADA2 (v1.3.0 [37]), and the GTDB reference database (release: R10-RS226-1 [38]). The obtained ASVs were analyzed and visualized in R (v4.5.1 [39]) and the plots created with the R package ‘ggplot2’ (v3.5.2 [40]). To account for contamination in 16S rRNA gene amplicon data often encountered with low biomass samples from the deep biosphere, control filters were sampled in the tunnel, frozen in liquid nitrogen, processed as described above, and the ASVs were removed using the Decontam R package (v1.30 [41]) from all samples prior to the analyses. In addition, sequences from genera identified as common contaminants in the Census for Deep Life [42] were also removed. Finally, one sample from the 2021 Tef1 biofilm sampling was removed as it only contained two ASVs.

Beta (between-sample) diversity was estimated using the Vegan package (v2.7–2 [43]) with principal coordinate analysis (PCoA) using default settings based on a Hellinger transformed Bray Curtis dissimilarity matrix of relative ASV abundances. Differences in beta diversity of the populations in each of the samples were tested for dispersion and then analyzed by Permutational Multivariate Analysis of Variance (PERMANOVA) using the Vegan package. In addition, pairwise Multivariate Analysis of Variance (MANOVA) also used Vegan to test differences between the complete biofilm communities between one- and four-years of incubation using RVAideMemoire (v0.9–83-12 [44]) on the Hellinger transformed values. Finally, comparisons of the transformed data for the top 20 biofilm families between one- and four-years of incubation were tested using Analysis of Variance (ANOVA) followed by Tukey’s post hoc analysis in Vegan. Due to the non-normal and compositional nature of the relative abundance data, differences in relative abundance between the one- and four-year biofilm communities were assessed using non-parametric Wilcoxon rank-sum tests (Mann–Whitney U tests) for each family followed by Benjamini–Hochberg correction to control false discovery rates. Effect sizes were calculated with the mean difference in relative abundance between years with positive and negative effects indicating an increase and decrease in mean relative abundance from year 1 to 4, respectively. Details of the various ANOVA, PERMANOVA, MANOVA, and Wilcoxon comparisons are provided in Supplemental Tables 4, 6, and 7. After the beta diversity was analyzed, the ASVs from the macadam samples prior to placing the rock in the containers were further bioinformatically redacted using the Decontam R package from the respective biofilm and planktonic cell communities to remove introduced populations.

Bioinformatic prediction of metabolism in the biofilm and planktonic samples was performed using PICRUSt 2.0 [45] as part of the Ampliseq pipeline with default settings. The PICRUSt2 identified 99.9% of the input sequences with a mean nearest sequenced taxon index (NSTI) value of 1.1 ± 5.6 suggesting moderate confidence in the representation of community members by the searched reference genomes. The output was searched for genes of interest related to microbial metabolisms previously identified at the Äspö HRL (Supplemental Table 3).

## Results and Discussion

### Groundwater Geochemistry

**Fig. 2:**
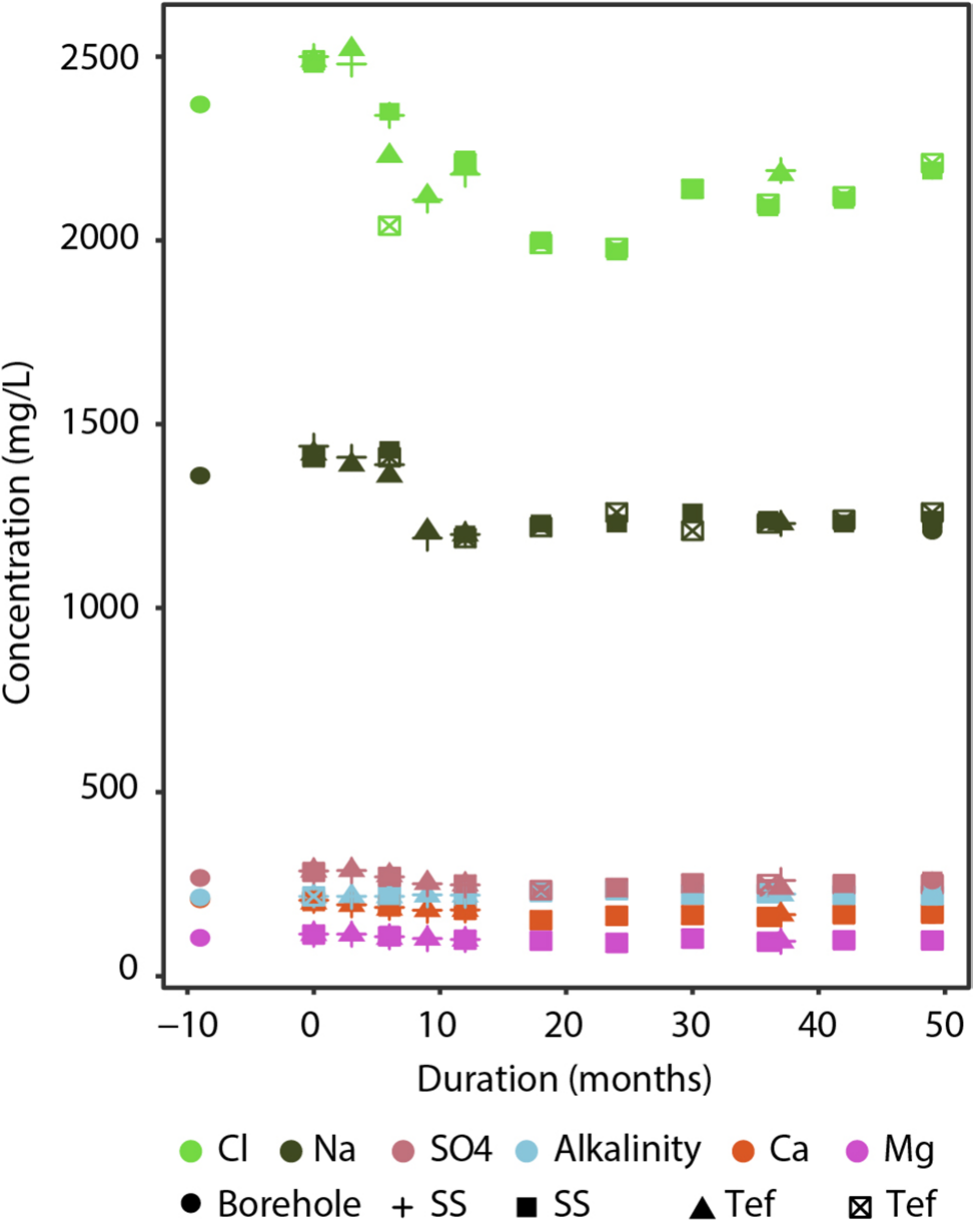
Time series of selected chemical analyses from the borehole before the experiment was carried out (−9 months) and after the experiment (48 months) along with the outflow from the four containers. The full data set is provided in Supplemental Table 2

Based upon the Cl and O^18^ variables, the HA0760 borehole intersected a fracture system bearing a ‘modern marine’ groundwater [30]. Most major elements and other variables in the groundwater itself along with the water flowing through the container outlets showed little variation from nine months prior to the experimental start date and the end point after 49 months incubation (Fig. 2 with details in Supplemental Table 2). In addition, the pH varied between 7.33 and 7.67 while the temperature varied between 10.4 °C in the borehole prior to the experiment to a maximum of 12.7 °C in container SS1 after six months due to the slightly higher value in the tunnel. However, the salinity (Na and Cl) decreased with time that may have several explanations including normal variation within analytical limits, disturbed or active rock surfaces releasing ions at the beginning of the experiment before an equilibrium was reached, or the groundwater being diluted by mixing of a less saline meteoric groundwater. The little variation in the physiochemical parameters reflected the stable conditions in deep Fennoscandian Shield groundwaters and likely did not strongly influence shifts in the container biofilm and planktonic microbial communities.

### 16S rRNA Gene-Based Beta Diversity

**Fig. 3:**
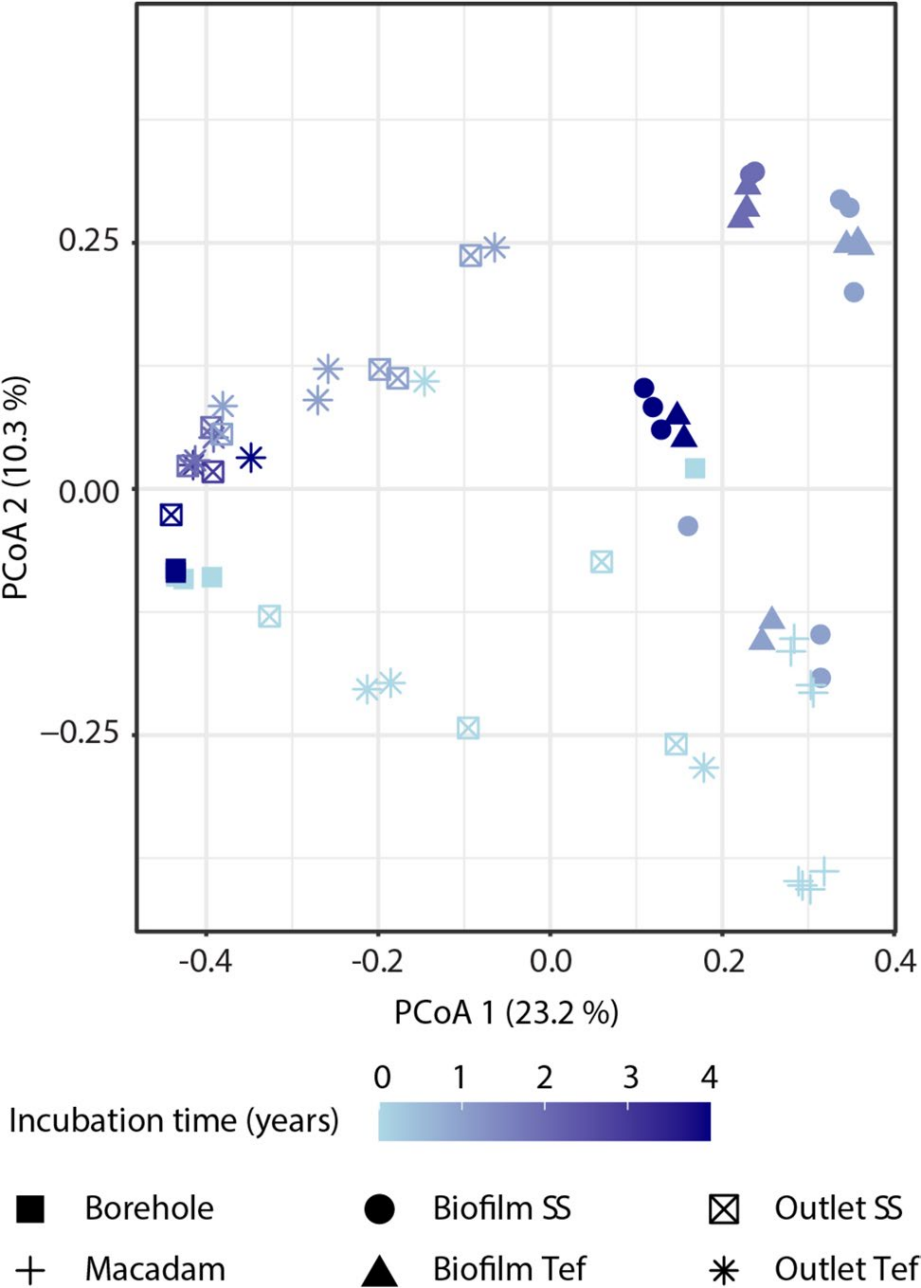
Beta diversity of the 16S rRNA gene-based communities for the groundwaters (sampled prior to and after the experiment), macadam used for biofilm formation, outflow water with planktonic cell communities from the four containers, and biofilms on the macadam surfaces. The figure is organized based upon the incubation time in years according to the study flow diagram in Supplemental Fig. 2 and supporting statistics in Supplemental Table 4

Beta diversity analysis of the borehole groundwater, macadam prior to the incubations, outlet water from the four containers, and biofilm on the macadam surface samples is provided in Fig. 3. PERMANOVA analysis of the differences in beta diversity of the populations between all sample types and incubation years showed a statistical significance (*p* < 0.001; details in Supplemental Table 4). The borehole water sampled without passing through the containers prior to and after the experiment clustered together with one exception while the MANOVA supported the community entering the containers did not alter significantly between December 2020 and April 2025 (*p* = 0.407 for combined SS and Tef containers; Supplemental Table 4). This supports the concept of a stable and slow growing deep biosphere community [14]. In contrast, the indigenous macadam community differed on the second axis between years, likely due to its being stored outside between its first use in the containers and addition after one and two years of storage. Furthermore, the planktonic populations in the Tef and SS container outlet waters generally became more similar to the natural groundwater with increasing incubation time (*p* = 0.046 and 0.360 after 0 and 4 years; Supplemental Table 4), potentially as the effect of the population added with the macadam was washed from the containers.

The biofilm communities after one year of incubation in the containers likewise shifted on the second axis forming two clusters based upon the first and second replicate one year incubation (Fig. 3), also potentially due to the changes in macadam community being stored outside as well as an initial colonization of attached populations on the natural rock surface. Furthermore, the biofilm communities after all incubation times were different to the ASVs introduced to the containers on the macadam surface when the incubations were initiated (all pairwise *p*-values ≤ 0.004; Supplemental Table 4). The biofilm community shifted with incubation time with all comparisons between biofilm incubation years showing significant differences (all pairwise *p*-values ≤ 0.020; Supplemental Table 4) suggesting the biofilm community evolved with time.

### 16S rRNA Gene-Based Groundwater Community

**Fig. 4:**
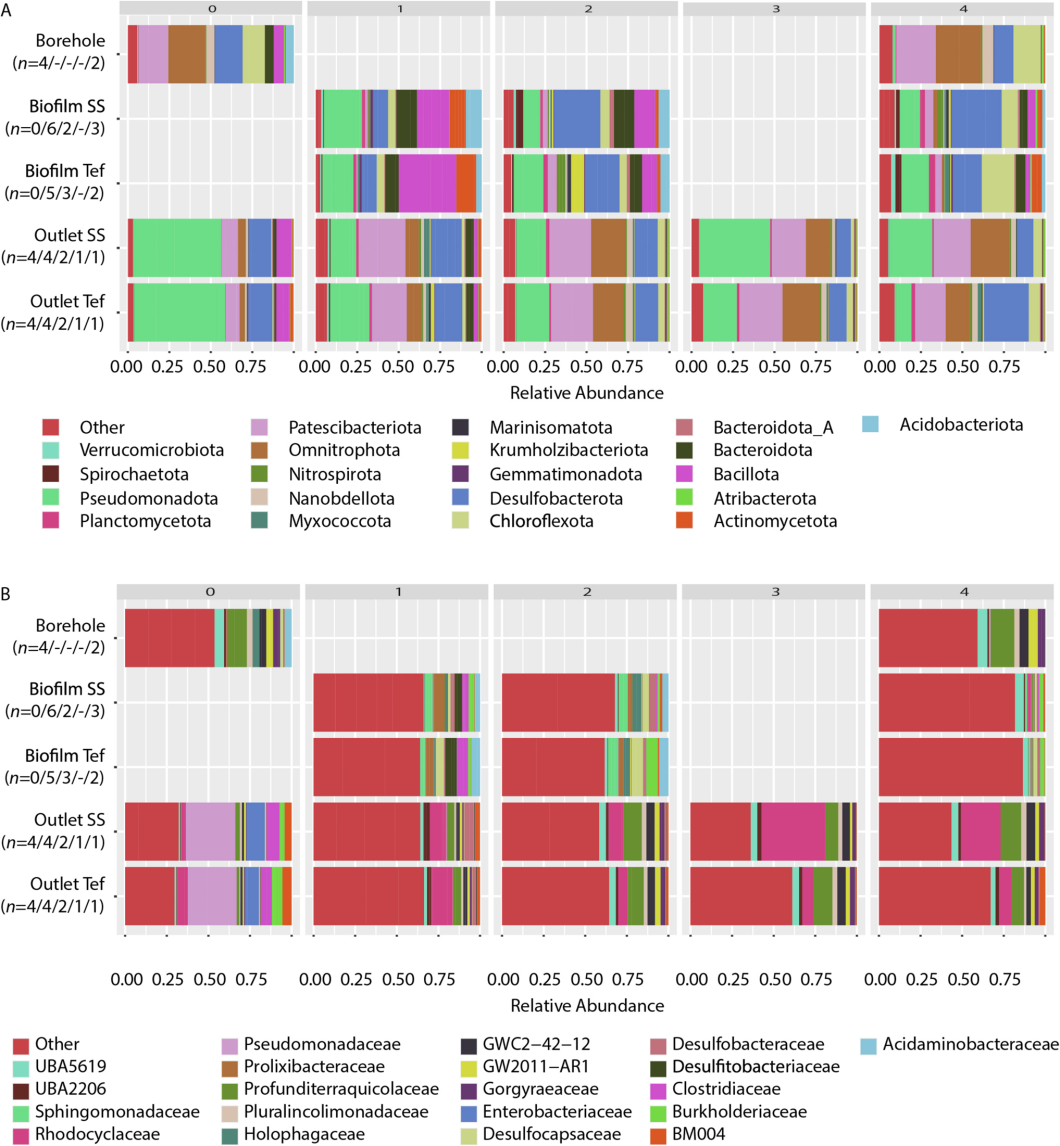
Stacked bar graph of 16S rRNA gene-based microbial communities at the phyla (**A**) and family (**B**) levels from the borehole groundwater, biofilm on the macadam surface, and outlet water from the stainless steel and Teflon lined containers. To show the biofilm specific 16S rRNA gene-based families, ASVs from the macadam were subtracted at each time point. The figure is organized based upon the incubation time in years according to the study flow diagram in Supplemental Fig. 2 and the top 20 taxa (based upon their relative abundance in all borehole, biofilm, and outlet samples analyzed after the indigenous macadam ASVs had been removed) are given with the remaining sequenced grouped into “other”. The families are read from left to right with “Other” first and then reverse alphabetical order. The number of replicates (*n*) is given for each sample and incubation year

Prior to initiation of the experiment, the families with the highest relative abundance in the groundwater (valves 1–4) were Profunditerraquicolaceae (Omnitrophota phylum; 11.3 ± 7.6), UBA5619 (Desulfobacterota; 5.7 ± 2.0), Acidaminobacteraceae (Bacillota; 4.3 ± 8.7), GW2011−AR1 (unclassified Archaea; 4.2 ± 3.6), GWC2−42−12 (Patescibacteria; 4.1 ± 2.7), Gorgyraeaceae (Omnitrophota; 4.1 ± 2.9), Holophagaceae (Acidobacteriota; 4.1 ± 8.3), Pluralincolimonadaceae (Omnitrophota; 3.5 ± 2.4), and Desulfocapsaceae (Desulfobacterota; 1.9 ± 3.7) (Fig. 4 with values in Supplemental Table 5 and individual stack bars for all samples in Supplemental Fig. 4). However, the valve four community had a different pattern and was dominated by Acidaminobacteraceae, Desulfocapsaceae, UBA5619, Prolixibacteraceae (Bacteroidota), and BM004 (Desulfobacterota). Profunditerraquicolaceae, Pluralincolimonadaceae, and Gorgyraeaceae are often host associated ultra-small cells [46], Acidaminobacteraceae contains a single described species isolated from estuary mud [47], and Desulfocapsaceae have been identified in anoxic deep Fennoscandian Shield groundwaters [48]. In addition, UBA5619 is an unclassified Syntrophaceae identified in groundwater, and GW2011−AR1 is also from groundwater. It was unknown why the valve four community was different, but its closer similarity to the biofilm communities on the beta diversity (Fig. 3) and stack bars (Supplemental Fig. 4) suggested detached biofilm cells from the Fennoscandian Shield rock inside of the borehole may have been captured in this sample.

### 16S rRNA Gene Macadam, Biofilm, and Planktonic Communities

Due to pairwise MANOVAs on the beta diversity showing no significant differences between the Tef and SS biofilm communities (all pairwise *p*-values ≥ 0.111; Supplemental Table 6), the data are discussed irrespective of the container lining. The macadam samples (Supplemental Fig. 4) contained ASVs assigned to families in common with the Tef and SS replicate biofilm 16S rRNA gene ASV profiles (Fig. 4 with biofilm values in Supplemental Table 5). These populations included Burkholderiaceae, Sphingomonadaceae, and Xanthomonadaceae (all three from the Pseudomonadota) and were not identified in the groundwaters and thus, were likely the indigenous populations present on the unsterilized macadam. In addition, the *Nocardioides* genus (family Nocardioidaceae, phylum Actinomycetota) was identified in both the Tef and SS biofilms (outside of the top 20 genera). This family was also present in drill water used when boring at the Äspö HRL [21] that coupled with its aerobic growth, suggested it was likely a surface taxon. This influence of the autochthonous macadam community highlights the care needed when sampling the deep biosphere as small amounts of DNA from surface related taxa can influence the conclusions [49].

To show the biofilm specific 16S rRNA gene-based families, the ASVs introduced by the macadam were subtracted from the biofilm community at each time point (Fig. 4 and Supplemental Table 5 and additional taxonomic levels in Supplemental Fig. 5). A PERMANOVA analysis of 16S rRNA gene biofilm ASVs between one- and four-year incubations also showed the community had changed with time (*p* = 0.001; Supplemental Table 7), further supporting the beta diversity analysis of an evolving biofilm community. The one-year incubation biofilm 16S rRNA gene-based families with highest relative abundances not also identified on the macadam included the Desulfitobacteriaceae (Bacillota), Desulfocapsaceae, and Desulfobacteraceae (Desulfobacterota) with average relative abundances of 5.6 ± 6.4, 3.1 ± 3.0, and 1.7 ± 1.8%, respectively. Of the Desulfitobacteriaceae, the majority of ASVs aligned with the sulfur reducing *Desulfosporosinus* genus (5.6 ± 6.4%) that is enriched in rock attached populations in the continental sub-surface [50], the Desulfocapsaceae are present in the deep terrestrial subsurface [48], and Desulfobacteraceae that predominantly encompassed ASVs aligning with the *Desulfobacula* genus and comprising 1.6 ± 1.7% of the biofilm relative abundance. Over time, the Desulfitobacteriaceae were only identified in the top 20 families after one year of incubation while the Desulfocapsaceae (5.8 ± 1.8 and 1.8 ± 0.8%) and Desulfobacteraceae (2.5 ± 1.6 and 0.7 ± 0.3) remained within the top 20 families after two and four years, respectively. In addition, the UBA5619 family increased in relative abundance with biofilm incubation from zero- to four-years including (0.2 ± 0.1 to 4.3 ± 1.6%; statistical support provided in Supplemental Table 7) that is present in Äspö HRL biofilms but not the planktonic fraction [31]. In contrast, the families Prolixibacteraceae, Clostridiaceae (Bacillota), Acidaminobacteraceae, and Sphingomonadaceae decreased from 5.6 ± 6.2, 5.2 ± 4.2, 4.3 ± 3.8, and 4.0 ± 2.7% after one year to 0.2 ± 0.1, 0.7 ± 0.5, 0.2 ± 0.2, and 1.2 ± 0.4%, respectively after four years incubation. Previous identification of biofilm populations in the deep biosphere includes an isolate from Horonobe, Hokkaido, Japan deep groundwater of assigned to the Prolixibacteraceae [51] and Clostridiales containing the Clostridiaceae from Fennoscandian rock surfaces [27]. The decrease in relative abundance of the Prolixibacteraceae and Acidaminobacteraceae potentially reflected their presence in the groundwater planktonic population that had a lessening effect on the biofilm community with increasing incubation time on the macadam surfaces. Finally, the BM004, Rhodocyclaceae (Pseudomonadota), Profunditerraquicolaceae, and UBA2206 (Patescibacteriota) families were present with no statistically significant changes in the biofilm communities with maximum relative abundances of 1.1 ± 0.4, 1.7 ± 1.0, 0.8 ± 0.5, and 0.4 ± 0.5%, respectively. The BM004 family (Desulfobacterota) is from the order Desulfobulbales that mostly encompasses sulfate reducing bacteria [52] and along with UBA2206 have been identified in groundwaters [53, 54].

Additional analysis of the 20 families with the highest relative abundance in the biofilms only without including the other samples (Supplemental Fig. 6) identified Acholeplasmataceae (Bacillota) and Geobacteraceae (Desulfobacterota) that had lower but consistently present relative abundances. The Acholeplasmataceae are present in Äspö HRL groundwaters (but not in the dominating biofilm families) [32] and the Outokumpu Deep Drill Hole [27] while Geobacteraceae ASVs assigned to *Geobacter* and *Geothrix* constitute ≤ 20% of a pristine aquifer biofilm community but ≤ 1% in the planktonic community [22]. Furthermore, the Anaerolineaceae (Chloroflexota) and UBA9217 (Nitrospirota) families increased their relative abundance within the biofilm communities after four years (statistical support in Supplemental Table 7). As for Acholeplasmataceae, the Anaerolineaceae are present in Äspö HRL groundwaters but not in the dominating biofilm families [32] and UBA9217 is most often identified from the marine and terrestrial deep biospheres [55]. Previous studies of Äspö HRL biofilms on glass and garnet surfaces identified differing populations such as hydrogen and inorganic sulfur oxidizing *Sulfurimonas* [31] and inorganic sulfur oxidizing *Thiobacillus* genera [32]. However, it is unknown if this is because the earlier studies used differing solid matrices or the longer incubation times in this study allowed for a mature biofilm to form.

Biofilm and outlet communities were searched for global deep terrestrial biosphere phyla [56] with streamlined genomes (genomes size < 1.6 Mb [57]; data and statistics in Supplemental Table 8). The 16S rRNA gene-based biofilm communities comprised 1.16 and 4.63% of the respective relative abundance of streamlined genome taxa after one- and four-years incubation compared to 24.6 and 20.7% in the outlet water. The one-year incubation streamlined genome taxa in the biofilm community solely comprised of Patescibacteriota with 1.16% that increased to 4.61% after four years of incubation (statistics in Supplemental Table 8) along with Aenigmatarchaeota, B1Sed10-29, Hydrothermarchaeota, Iainarchaeota, and Micrarchaeota. Due to the low number of identified ASVs attributed to taxa with streamlined genomes at one-year of incubation, only the increase in Patescibacteriota could be statistically tested and showed a *p* value of 0.002. The outlet water streamlined genome taxa after one- and four-years of incubation was also dominated by Patescibacteriota (24.5 & 20.3%, respectively) together with lesser relative abundances of the phyla present in the biofilm along with Hadarchaeota and Undinarchaeota. This supported a previous hypothesis that populations present in groundwater such as those with streamlined genomes and a limited metabolic potential might join the biofilm once it had matured [32]. The contrasting deep biosphere biofilm versus planktonic families in this study compared to other Fennoscandian Shield communities highlights factors such as incubation time, differing communities between groundwaters, and the solid surface that the cells attach to having an important influence on the biofilm community [21, 27, 31, 32].

### Metabolic Prediction of Biofilm Populations

**Fig. 5:**
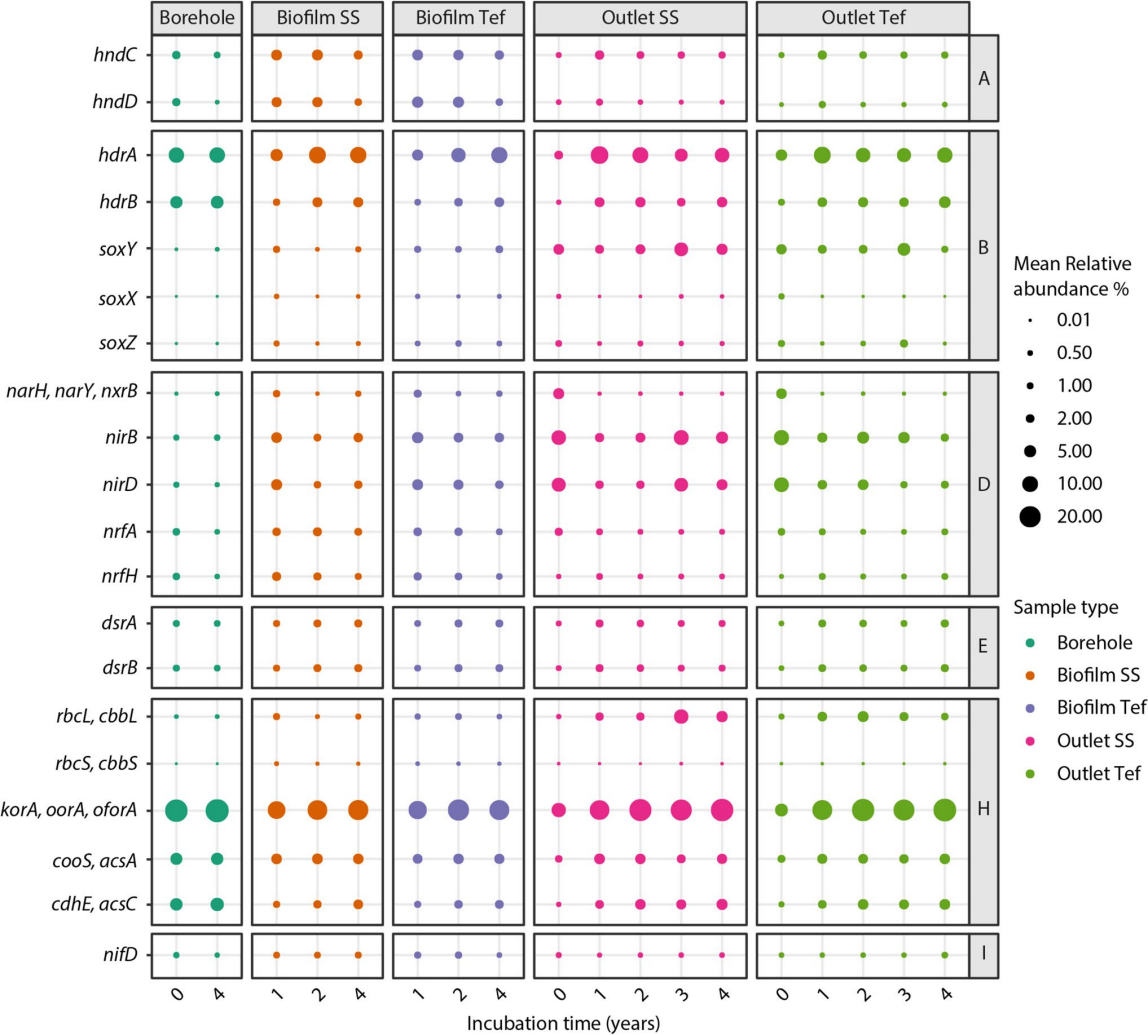
Metabolic prediction based upon the 16S rRNA gene amplicon abundances for selected genes (full analysis presented in Supplemental Fig. 7). Abbreviations: A, hydrogen oxidation; B, sulfur oxidation; D, dissimilatory nitrate reduction; E, dissimilatory sulfate reduction; H, carbon fixation; and I, nitrogen fixation

As the four-year biofilms were most distinct from the macadam samples, they were analyzed for 16S rRNA gene-based metabolic characteristics using PICRUSt2 (Fig. 5 with searched genes in Supplemental Table 2 and the full data in Supplemental Fig. 7). The four-year biofilm community was predicted to fix carbon dioxide via pathways including the reverse TCA cycle (e.g., *korA, oorA, oforA*), the Wood-Ljungdahl pathway (e.g., *cooS*, *acsA*, *cdhE*, *acsC*), and the Calvin-Benson-Bassham (CBB) cycle (*rbcLS*, *cbbLS*) and to fix nitrogen via the *nif* genes (e.g., *nifD*). Carbon sources in the terrestrial deep biosphere include carbon dioxide as geogases [58], necromass from dead cells that infiltrate from the surface but are unable to survive [15], and terrigenous dissolved organic matter from the surface [59]. To support growth, the predicted electron donors for the biofilm community included hydrogen via anaerobic NADP-dependent hydrogenases (*hndCD*). Further predicted biofilm electron donors were sulfur using genes coding for heterodisulfide reductase (*hdrAB*) and inorganic sulfur compound oxidation using a partial SOX complex (*soxXYZ*). These electron donors were predicted to be coupled with e.g., dissimilatory nitrate reduction (e.g., *narHY*, *nxrB*), denitrification (e.g., *nirBD*, *nrfAH*), and sulfate reduction (e.g., *dsrAB*). The predicted biofilm metabolic pathways were consistent with the deep terrestrial biosphere community where sulfur cycling is active in Fennoscandian Shield groundwaters [60]. In addition, the metabolic pathways in this long-term biofilm study matched previous analysis of early biofilm formation in deep Fennoscandian groundwaters where the communities were dominated by carbon and nitrogen fixing populations with hydrogen and/or sulfur cycling as the major mode of energy conservation [31, 32]. In addition, comparison of the metabolic predictions for these deep groundwater biofilms compared to a shallow, 48 m deep aquifer suggested similar results in oxic and anoxic carbonate rocks from the Hainich Critical Zone Exploratory with biofilms being dominated by chemolithoautotrophic populations cycling e.g., iron and sulfur [61]. Despite the differing rock matrix, this suggested a partial consistency between shallow and deep groundwater biofilm metabolisms.

## Conclusions

The results supported the first part of the hypothesis that the biofilm and planktonic communities were distinct while the second part of the hypothesis was partially supported as while populations predicted to be ultra-small and with streamlined genomes were identified in the one-year-old biofilm, they increased in relative abundance with time. Finally, in contrast to previous studies, the attached and planktonic population metabolisms were more consistent, suggesting multi-year biofilms become more metabolically heterogenous as compared to early biofilm formation that is dominated by a fewer number of autotrophic and diazotrophic founder populations.

## Supplementary Information

Below is the link to the electronic supplementary material.

Supplementary file1 (DOCX 16757 KB)

## Data Availability

16S rRNA gene sequences are available under BioProject accession number PRJEB105326 and sample accession numbers ERS28332009-ERS28332075. The R markdown used to generate the figures is at https://github.com/AndersCJohnson/AndersCJohnson/tree/Aspo\_biofilm\_clogging\_experiement.

## References

[1] Gold T (1992) The deep, hot biosphere. Proc Natl Acad Sci U S A 89:6045–6049. 10.1073/pnas.89.13.6045 1631089 10.1073/pnas.89.13.6045 PMC49434

[2] Magnabosco C, Lin LH, Dong H, Bomberg M, Ghiorse W, Stan-Lotter H, Pedersen K, Kieft TL, van Heerden E, Onstott TC (2018) The biomass and biodiversity of the continental subsurface. Nat Geosci 11:707–717. 10.1038/s41561-018-0221-6

[3] Drake H, Reiners PW (2021) Thermochronologic perspectives on the deep-time evolution of the deep biosphere. Proc Natl Acad Sci U S A 118:e2109609118. 10.1073/pnas.2109609118 34725158 10.1073/pnas.2109609118 PMC8609299

[4] Bar-On YM, Phillips R, Milo R (2018) The biomass distribution on Earth. Proc Natl Acad Sci U S A 115:6506–6511. 10.1073/pnas.1711842115 29784790 10.1073/pnas.1711842115 PMC6016768

[5] Beaver RC, Neufeld JD (2024) Microbial ecology of the deep terrestrial subsurface. ISME J 18. 10.1093/ismejo/wrae1091 10.1093/ismejo/wrae091 PMC11170664 38780093

[6] Cai L, Weinbauer MG, Xie L, Zhang R (2023) The smallest in the deepest: the enigmatic role of viruses in the deep biosphere. Natl Sci Rev 10. 10.1093/nsr/nwad1009 10.1093/nsr/nwad009 PMC10029852 36960220

[7] Dopson M, Somee MR, González-Rosales C, Lui LM, Turner S, Buck M, Nilsson E, Westmeijer G, Ashoor K, Nielsen TN, Mehrshad M, Bertilsson S (2024) Novel candidate taxa contribute to key metabolic processes in Fennoscandian Shield deep groundwaters. ISME Commun 4:ycae113. 10.1093/ismeco/ycae113 39421601 10.1093/ismeco/ycae113 PMC11484514

[8] Wu X, Holmfeldt K, Hubalek V, Lundin D, Åström M, Bertilsson S, Dopson M (2016) Microbial metagenomes from three aquifers in the Fennoscandian shield terrestrial deep biosphere reveal metabolic partitioning among populations. ISME J 10:1192–1203. 10.1038/ismej.2015.185 26484735 10.1038/ismej.2015.185 PMC5029217

[9] Nelson WC, Stegen JC (2015) The reduced genomes of *Parcubacteria* (OD1) contain signatures of a symbiotic lifestyle. Front Microbiol 6. 10.3389/fmicb.2015.00713 10.3389/fmicb.2015.00713 PMC4508563 26257709

[10] Tian R, Ning D, He Z, Zhang P, Spencer SJ, Gao S, Shi W, Wu L, Zhang Y, Yang Y, Adams BG, Rocha AM, Detienne BL, Lowe KA, Joyner DC, Klingeman DM, Arkin AP, Fields MW, Hazen TC, Stahl DA, Alm EJ, Zhou J (2020) Small and mighty: adaptation of superphylum Patescibacteria to groundwater environment drives their genome simplicity. Microbiome 8:51. 10.1186/s40168-020-00825-w 32252814 10.1186/s40168-020-00825-w PMC7137472

[11] He C, Keren R, Whittaker ML, Farag IF, Doudna JA, Cate JHD, Banfield JF (2021) Genome-resolved metagenomics reveals site-specific diversity of episymbiotic CPR bacteria and DPANN archaea in groundwater ecosystems. Nat Microbiol 6:354–365. 10.1038/s41564-41020-00840-41565 33495623 10.1038/s41564-020-00840-5 PMC7906910

[12] Castelle CJ, Brown CT, Anantharaman K, Probst AJ, Huang RH, Banfield JF (2018) Biosynthetic capacity, metabolic variety and unusual biology in the CPR and DPANN radiations. Nat Rev Microbiol 16:629–645. 10.1038/s41579-018-0076-2 30181663 10.1038/s41579-018-0076-2

[13] Herrmann M, Wegner C-E, Taubert M, Geesink P, Lehmann K, Yan L, Lehmann R, Totsche KU, Küsel K (2019) Predominance of *Cand.* Patescibacteria in groundwater is caused by their preferential mobilization from soils and flourishing under oligotrophic conditions. Front Microbiol 10:1407. 10.3389/fmicb.2019.01407 10.3389/fmicb.2019.01407 PMC6596338 31281301

[14] Lloyd KG, Steen AD (2025) Defining ultra-slow-growing extremophilic microorganisms as aeonophiles. Nat Microbiol 10:1555–1557. 10.1038/s41564-025-02048-x 40542286 10.1038/s41564-025-02048-x

[15] Mehrshad M, Lopez-Fernandez M, Sundh J, Bell E, Simone D, Buck M, Bernier-Latmani R, Bertilsson S, Dopson M (2021) Energy efficiency and biological interactions define the core microbiome of deep oligotrophic groundwater. Nat Commun 12:4253. 10.1038/s41467-021-24549-z 10.1038/s41467-021-24549-z PMC8275790 34253732

[16] Lloyd JR, Cherkouk A (2021) The microbiology of nuclear waste disposal. Elsevier, Amsterdam, Netherlands

[17] Dopffel N, Jansen S, Gerritse J (2021) Microbial side effects of underground hydrogen storage – knowledge gaps, risks and opportunities for successful implementation. Int J Hydrog Energy 46:8594–8606. 10.1016/j.ijhydene.2020.12.058

[18] Flemming HC, Wingender J, Szewzyk U, Steinberg P, Rice SA, Kjelleberg S (2016) Biofilms: an emergent form of bacterial life. Nat Rev Microbiol 14:563–575. 10.1038/nrmicro.2016.94 27510863 10.1038/nrmicro.2016.94

[19] Watnick P, Kolter R (2000) Biofilm, City of Microbes. J Bacteriol 182:2675–2679. 10.1128/jb.182.10.2675-2679.2000 10781532 10.1128/jb.182.10.2675-2679.2000 PMC101960

[20] Flemming H-C, Wuertz S (2019) Bacteria and archaea on Earth and their abundance in biofilms. Nat Rev Microbiol 17:247–260. 10.1038/s41579-019-0158-9 30760902 10.1038/s41579-019-0158-9

[21] Jägevall S, Rabe L, Pedersen K (2011) Abundance and diversity of biofilms in natural and artificial aquifers of the Äspö Hard Rock Laboratory, Sweden. Microb Ecol 61:410–422. 10.1007/s00248-010-9761-z 21132427 10.1007/s00248-010-9761-z

[22] Flynn TM, Sanford RA, Bethke CM (2008) Attached and suspended microbial communities in a pristine confined aquifer. Water Resour Res 44:W07425. 10.1029/2007WR006633

[23] Casar CP, Kruger BR, Flynn TM, Masterson AL, Momper LM, Osburn MR (2020) Mineral-hosted biofilm communities in the continental deep subsurface, Deep Mine Microbial Observatory, SD, USA. Geobiology 18:508–522. 10.1111/gbi.12391 32216092 10.1111/gbi.12391

[24] Uroz S, Calvaruso C, Turpault M-P, Frey-Klett P (2009) Mineral weathering by bacteria: ecology, actors and mechanisms. Trends Microbiol 17:378–387. 10.1016/j.tim.2009.05.004 19660952 10.1016/j.tim.2009.05.004

[25] Gleeson DB, Kennedy NM, Clipson N, Melville K, Gadd GM, McDermott FP (2006) Characterization of bacterial community structure on a weathered pegmatitic granite. Microb Ecol 51:526–534. 10.1007/s00248-006-9052-x 16649062 10.1007/s00248-006-9052-x

[26] Amano Y, Iwatsuki T, Naganuma T (2017) Characteristics of naturally grown biofilms in deep groundwaters and their heavy metal sorption property in a deep subsurface environment. Geomicrobiol J 34:769–783. 10.1080/01490451.2016.1267281

[27] Nuppunen-Puputti M, Kietäväinen R, Purkamo L, Rajala P, Itävaara M, Kukkonen I, Bomberg M (2021) Rock surface fungi in deep continental biosphere—exploration of microbial community formation with subsurface *in situ* biofilm trap. Microorganisms 9:64 10.3390/microorganisms9010064 PMC7824546 33383728

[28] Mathurin FA, Astrom ME, Laaksoharju M, Kalinowski BE, Tullborg EL (2012) Effect of tunnel excavation on source and mixing of groundwater in a coastal granitoidic fracture network. Environ Sci Technol 46:12779–12786. 10.1021/es301722b 23088667 10.1021/es301722b

[29] Lopez-Fernandez M, Broman E, Wu X, Bertilsson S, Dopson M (2018) Investigation of viable taxa in the deep terrestrial biosphere suggests high rates of nutrient recycling. FEMS Microbiol Ecol 94. 10.1093/femsec/fiy1121 10.1093/femsec/fiy121 PMC6030916 29931252

[30] Lopez-Fernandez M, Åström M, Bertilsson S, Dopson M (2018) Depth and dissolved organic carbon shape microbial communities in surface influenced but not ancient saline terrestrial aquifers. Front Microbiol 9:2880 30538690 10.3389/fmicb.2018.02880 PMC6277548

[31] Wu X, Pedersen K, Edlund J, Eriksson L, Åström M, Anderson AF, Bertilsson S, Dopson M (2017) Potential for hydrogen-oxidizing chemolithoautotrophic and diazotrophic populations to initiate biofilm formation in oligotrophic, deep terrestrial subsurface waters. Microbiome 5:35. 10.1186/s40168-40017-40253-y 10.1186/s40168-017-0253-y PMC5364579 28335808

[32] Lopez-Fernandez M, Westmeijer G, Turner S, Broman E, Ståhle M, Bertilsson S, Dopson M (2023) *Thiobacillus* as a key player for biofilm formation in oligotrophic groundwaters of the Fennoscandian Shield. npj Biofilms Microbiomes 9(1):41. 10.1038/s41522-023-00408-1 10.1038/s41522-023-00408-1 PMC10287647 37349512

[33] Nilsson A-C, Gimeno MJ, Tullborg E-L, Mathurin F, Smellie J (2013) Hydrogeochemical data report Äspö Site descriptive modelling Äspö SDM. SKB R-13–26 Svensk Kärnbränslehantering AB

[34] Hugerth LW, Wefer HA, Lundin S, Jakobsson HE, Lindberg M, Rodin S, Engstrand L, Andersson AF (2014) DegePrime, a program for degenerate primer design for broad-taxonomic-range PCR in microbial ecology studies. Appl Environ Microbiol 80:5116–5123. 10.1128/aem.01403-14 24928874 10.1128/AEM.01403-14 PMC4135748

[35] Herlemann DP, Labrenz M, Jurgens K, Bertilsson S, Waniek JJ, Andersson AF (2011) Transitions in bacterial communities along the 2000 km salinity gradient of the Baltic Sea. ISME J 5:1571–1579. 10.1038/ismej.2011.41 21472016 10.1038/ismej.2011.41 PMC3176514

[36] Straub D, Blackwell N, Langarica-Fuentes A, Peltzer A, Nahnsen S, Kleindienst S (2020) Interpretations of environmental microbial community studies are biased by the selected 16S rRNA (gene) amplicon sequencing pipeline. Front Microbiol 11. 10.3389/fmicb.2020.550420 10.3389/fmicb.2020.550420 PMC7645116 33193131

[37] Callahan BJ, McMurdie PJ, Rosen MJ, Han AW, Johnson AJA, Holmes SP (2016) DADA2: high-resolution sample inference from Illumina amplicon data. Nat Methods 13:581. 10.1038/nmeth.3869 27214047 10.1038/nmeth.3869 PMC4927377

[38] Chaumeil P-A, Mussig AJ, Hugenholtz P, Parks DH (2019) GTDB-Tk: a toolkit to classify genomes with the Genome Taxonomy Database. Bioinformatics. 10.1093/bioinformatics/btz848 31730192 10.1093/bioinformatics/btz848 PMC7703759

[39] R Core Team (2025). R: A Language and Environment for Statistical Computing. R Foundation for Statistical Computing, Vienna, Austria. R version 4.5.2. ISBN: 3–900051–07–0: http://www.R-project.org/

[40] Wickham H (2016) ggplot2: Elegant Graphics for Data Analysis. Springer-Verlag, New York

[41] Davis NM, Proctor DM, Holmes SP, Relman DA, Callahan BJ (2018) Simple statistical identification and removal of contaminant sequences in marker-gene and metagenomics data. Microbiome 6:226. 10.1186/s40168-018-0605-2 30558668 10.1186/s40168-018-0605-2 PMC6298009

[42] Sheik CS, Reese BK, Twing KI, Sylvan JB, Grim SL, Schrenk MO, Sogin ML, Colwell FS (2018) Identification and removal of contaminant sequences from ribosomal gene databases: Lessons from the census of deep life. Front Microbiol 9:00840. 10.3389/fmicb.2018.00840 10.3389/fmicb.2018.00840 PMC5945997 29780369

[43] Oksanen J, L. SG, Guillaume Blanchet F, Kindt R, Legendre P, Minchin PR, O'Hara RB, Solymos P, Stevens MHH, Szoecs E, Wagner H, Barbour M, Bedward M, Bolker B, Borcard D, Borman T, Carvalho G, Chirico M, De Caceres M, Durand S, Evangelista HBA, FitzJohn R, Friendly M, Furneaux B, Hannigan G, Hill MO, Lahti L, Martino C, McGlinn D, Ouellette M-H, Ribeiro Cunha E, Smith T, Stier A, Ter Braak CJF, Weedon J (2025) Vegan: community ecology package. R Package v2.7-2. 10.32614/CRAN.package.vegan

[44] Herve M (2025) RVAideMemoire: testing and plotting procedures for biostatistics. R package version 205 0.9–83–12. 10.32614/CRAN.package.RVAideMemoire

[45] Douglas GM, Maffei VJ, Zaneveld JR, Yurgel SN, Brown JR, Taylor CM, Huttenhower C, Langille MGI (2020) PICRUSt2 for prediction of metagenome functions. Nat Biotechnol 38:685–688. 10.1038/s41587-020-0548-6 32483366 10.1038/s41587-020-0548-6 PMC7365738

[46] Seymour CO, Palmer M, Becraft ED, Stepanauskas R, Friel AD, Schulz F, Woyke T, Eloe-Fadrosh E, Lai D, Jiao J-Y, Hua Z-S, Liu L, Lian Z-H, Li W-J, Chuvochina M, Finley BK, Koch BJ, Schwartz E, Dijkstra P, Moser DP, Hungate BA, Hedlund BP (2023) Hyperactive nanobacteria with host-dependent traits pervade Omnitrophota. Nat Microbiol 8:727–744. 10.1038/s41564-022-01319-1 36928026 10.1038/s41564-022-01319-1 PMC10066038

[47] Stams AJM, Hansen TA (1984) Fermentation of glutamate and other compounds by *Acidaminobacter hydrogenoformans* gen. nov. sp. nov., an obligate anaerobe isolated from black mud. Studies with pure cultures and mixed cultures with sulfate-reducing and methanogenic bacteria. Arch Microbiol 137:329–337. 10.1007/BF00410730

[48] Bell E, Lamminmäki T, Alneberg J, Qian C, Xiong W, Hettich RL, Frutschi M, Bernier-Latmani R (2022) Active anaerobic methane oxidation and sulfur disproportionation in the deep terrestrial subsurface. ISME J. 10.1038/s41396-41022-01207-w 35173296 10.1038/s41396-022-01207-w PMC9123182

[49] Westmeijer G, Escudero C, Bergin C, Turner S, Ståhle M, Mehrshad M, Leroy P, Buck M, López-Hernández P, Kallmeyer J, Amils R, Bertilsson S, Dopson M (2024) Continental scientific drilling and microbiology: (extremely) low biomass in crystalline bedrock of central Sweden. Biogeosciences 21:591–604. 10.5194/bg-21-591-2024

[50] Heinze BM, Schwab VF, Trumbore SE, Schroeter SA, Xu X, Chaudhari NM, Küsel K (2025) Old but not ancient: rock-leached organic carbon drives groundwater microbiomes. Sci Total Environ 959:178212. 10.1016/j.scitotenv.2024.178212 10.1016/j.scitotenv.2024.178212 39721524

[51] Ueno A, Sato K, Tamamura S, Murakami T, Inomata H, Tamazawa S, Amano Y, Miyakawa K, Naganuma T, Igarashi T (2025) *Gaoshiqia hydrogeniformans* sp. nov., a novel hydrogen-producing bacterium isolated from a deep diatomaceous shale formation. Int J Evol Syst Evol Microbiol 75. 10.1099/ijsem.0.006802 10.1099/ijsem.0.006802 40465470

[52] Ward LM, Bertran E, Johnston DT (2021) Expanded genomic sampling refines current understanding of the distribution and evolution of sulfur metabolisms in the Desulfobulbales. Front Microbiol 12:666052. 10.3389/fmicb.2021.666052 34093483 10.3389/fmicb.2021.666052 PMC8170396

[53] Anantharaman K, Brown CT, Hug LA, Sharon I, Castelle CJ, Probst AJ, Thomas BC, Singh A, Wilkins MJ, Karaoz U, Brodie EL, Williams KH, Hubbard SS, Banfield JF (2016) Thousands of microbial genomes shed light on interconnected biogeochemical processes in an aquifer system. Nat Commun 7:13219 10.1038/ncomms13219 PMC5079060 27774985

[54] Brown CT, Hug LA, Thomas BC, Sharon I, Castelle CJ, Singh A, Wilkins MJ, Wrighton KC, Williams KH, Banfield JF (2015) Unusual biology across a group comprising more than 15% of domain Bacteria. Nature 523:208–211. 10.1038/nature14486 26083755 10.1038/nature14486

[55] D’Angelo T, Goordial J, Lindsay MR, McGonigle J, Booker A, Moser D, Stepanauskus R, Orcutt BN (2023) Replicated life-history patterns and subsurface origins of the bacterial sister phyla Nitrospirota and Nitrospinota. ISME J 17:891–902. 10.1038/s41396-023-01397-x 37012337 10.1038/s41396-023-01397-x PMC10203281

[56] González-Rosales C, Rezaei Somee M, Buck M, Bertilsson S, Mehrshad M, Dopson M (2025) A global deep terrestrial biosphere core microbiome. ISME Commun 5:ycaf176. 10.1093/ismeco/ycaf176 41216320 10.1093/ismeco/ycaf176 PMC12596165

[57] Rodríguez-Gijón A, Pacheco-Valenciana A, Milke F, Dharamshi JE, Hampel JJ, Damashek J, Wienhausen G, Rodriguez-R LM, Garcia SL (2025) Widely-distributed freshwater microorganisms with streamlined genomes co-occur in cohorts with high abundance. Sci Rep 15:34482. 10.1038/s41598-025-22383-7 41044404 10.1038/s41598-025-22383-7 PMC12495000

[58] Lopez-Fernandez M, Broman E, Simone D, Bertilsson S, Dopson M (2019) Statistical analysis of community RNA transcripts between organic carbon and ‘geogas’ fed continental deep biosphere groundwaters. mBio 10(4):e01470-e1419. 10.1128/mbio.01470-19 31409677 10.1128/mBio.01470-19 PMC6692508

[59] Osterholz H, Turner S, Alakangas LJ, Tullborg E-L, Dittmar T, Kalinowski BE, Dopson M (2022) Terrigenous dissolved organic matter persists in the energy-limited deep groundwaters of the Fennoscandian Shield. Nat Commun 13:4837. 10.1038/s41467-022-32457-z 35977924 10.1038/s41467-022-32457-z PMC9385861

[60] Bell E, Lamminmäki T, Alneberg J, Andersson AF, Qian C, Xiong W, Hettich RL, Frutschi M, Bernier-Latmani R (2020) Active sulfur cycling in the terrestrial deep subsurface. ISME J 14:1260–1272. 10.1038/s41396-020-0602-x 32047278 10.1038/s41396-020-0602-x PMC7174417

[61] Sharma A, Küsel K, Wegner CE, Pérez-Carrascal OM, Taubert M (2025) Two worlds beneath: Distinct microbial strategies of the rock-attached and planktonic subsurface biosphere. Microbiome 14:73 10.1186/s40168-025-02325-1 PMC12930757 41620752

[62] Pedersen K, Arlinger J, Ekendahl S, Hallbeck L (1996) 16S rRNA gene diversity of attached and unattached bacteria in boreholes along the access tunnel to the Äspö hard rock laboratory, Sweden. FEMS Microbiol Ecol 6496:249–262. 10.1016/0168-6496(96)00017-7

