# Supplementary material for: Multi-Year Biofilm Formation on Granitic Surfaces Reveals Dynamic Microbial Communities in Fennoscandian Shield Deep Groundwaters
Source: Microb Ecol. 2026 Jun 19;89(1):131. doi: 10.1007/s00248-026-02812-4 (PMC13283197; doi:10.1007/s00248-026-02812-4)

**Multi-Year Biofilm Formation on Granitic Surfaces Reveals Dynamic Microbial Communities in Fennoscandian Shield Deep Groundwaters**

Magnus Ståhle, Anders Johnson, Stephanie Turner, Per Mårtensson,
Birgitta Kalinowski, and Mark Dopson

**Supplemental Material**

**Supplemental Table 1** Details of the groundwater samples taken before and during the experiment for 16S rRNA gene analyses.

| **Sample** | **Sequencing User ID** | **Sample types** | **Sampling date** | **DNA  (ng/µL)** | **Kreads** | **>=Q30 (%)** | **DADA2 input sequences** | **DADA2 filtered sequences** | **Sequencing depth** | **Unique ASVs** | **Sampling volume (L)** |
| --- | --- | --- | --- | --- | --- | --- | --- | --- | --- | --- | --- |
| P37057_1001 | HA0760B 01.12.2020 Valve 1 | Borehole | 2020-12-01 | 40.6 | 336.08 | 80.1 | 156436 | 81271 | 41841 | 1643 | 75 |
| P37057_1002 | HA0760B 01.12.2020 Valve 2 | Borehole | 2020-12-01 | 43.5 | 155.45 | 80.03 | 72470 | 37285 | 12841 | 652 | 76 |
| P37057_1003 | HA0760B 01.12.2020 Valve 3 | Borehole | 2020-12-01 | 23.2 | 298.74 | 80.56 | 138590 | 72428 | 45295 | 1779 | 40 |
| P37057_1004 | HA0760B 01.12.2020 Valve 4 | Borehole | 2020-12-01 | 46.3 | 2.41 | 77.42 | 1057 | 540 | 176 | 16 | 100 |
| P37057_1005 | Control filter 01.12.2020 | Control | 2020-12-01 | <0.05 | 08.12 | 79.72 | 142704 | 73348 | 48371 | 1789 | - |
| P37057_1006 | Container A1 2020.12.10 | Outlet_SS1 | 2020-12-10 | 53 | 230.56 | 80.75 | 108525 | 56598 | 28551 | 606 | 77 |
| P37057_1007 | Container A2 2020.12.10 | Outlet_SS2 | 2020-12-10 | 49.4 | 224.11 | 80.28 | 104158 | 53925 | 43206 | 872 | 50 |
| P37057_1008 | Container B1 2020.12.10 | Outlet_Tef1 | 2020-12-10 | 48.7 | 268.38 | 80.22 | 125612 | 65373 | 38501 | 711 | 43 |
| P37057_1009 | Container B2 2020.12.10 | Outlet_Tef2 | 2020-12-10 | 54 | 193.23 | 79.65 | 86571 | 44603 | 25070 | 478 | 36 |
| P37057_1010 | Control filter 2020.12.10 | Control | 2020-12-10 | <0.05 | 0.76 | 51.34 | 308 | 31 | 0 | 0 | - |
| P37057_1011 | Macadam Lysis buffer – 1 | Macadam | 2020-12-16 | 0.136 | 260.5 | 79.84 | 116724 | 60612 | 46654 | 926 | - |
| P37057_1012 | Macadam Lysis buffer – 2 | Macadam | 2020-12-16 | 0.095 | 193.43 | 78.97 | 86767 | 44274 | 39599 | 1066 | - |
| P37057_1013 | Clogging container A1 2021.12.06 | Outlet_SS1 | 2021-12-06 | >60 | 198.27 | 79.09 | 87745 | 44655 | 25197 | 605 | 113 |
| P37057_1014 | Clogging container A2 2021.12.06 | Outlet_SS2 | 2021-12-06 | >60 | 183.38 | 79.84 | 81656 | 42224 | 24996 | 744 | 112 |
| P37057_1015 | Clogging container B1 2021.12.06 | Outlet_Tef1 | 2021-12-06 | >60 | 220.15 | 79.75 | 97838 | 50540 | 30809 | 972 | 80 |
| P37057_1016 | Clogging container B2 2021.12.06 | Outlet_Tef2 | 2021-12-06 | >60 | 196.41 | 79.44 | 88030 | 45152 | 24533 | 687 | 89 |
| P37057_1017 | Control filter 2021.12.06 | Control | 2021-12-06 | <0.05 | 279.98 | 80.02 | 131552 | 67523 | 47415 | 449 | - |
| P37057_1018 | Clogging container A1 2021.12.20 | Outlet_SS1 | 2021-12-20 | >60 | 128.19 | 80.36 | 60759 | 31073 | 11083 | 202 | 68 |
| P37057_1019 | Clogging container B1 2021.12.20 | Outlet_Tef1 | 2021-12-20 | >60 | 265.07 | 80.6 | 123908 | 64553 | 24958 | 354 | 77 |
| P37057_1020 | Control filter 2021.12.20 | Control | 2021-12-20 | <0.05 | 247.54 | 79.39 | 114490 | 57942 | 53369 | 553 | - |
| P37057_1021 | Clogging macadam A1 End top 2021.12.14 | Biofilm_SS1 | 2021-12-14 | <0.05 | 470.16 | 77.26 | 219552 | 106518 | 100772 | 1029 | - |
| P37057_1022 | Clogging macadam A1 End middle 2021.12.14 | Biofilm_SS1 | 2021-12-14 | <0.05 | 65.64 | 79.95 | 30582 | 15864 | 14628 | 329 | - |
| P37057_1023 | Clogging macadam A1 End Bottom 2021.12.14 | Biofilm_SS1 | 2021-12-14 | <0.05 | 403.24 | 80.46 | 187139 | 97216 | 92514 | 959 | - |
| P37057_1024 | Clogging macadam B1 End top 2021.12.14 | Biofilm_Tef1 | 2021-12-14 | <0.05 | 0.26 | 63.34 | 89 | 28 | 2 | 1 | - |
| P37057_1025 | Clogging macadam B1 End middle 2021.12.14 | Biofilm_Tef1 | 2021-12-14 | <0.05 | 303.86 | 78.66 | 141314 | 71519 | 65591 | 1881 | - |
| P37057_1026 | Clogging macadam B1 End Bottom 2021.12.14 | Biofilm_Tef1 | 2021-12-14 | <0.05 | 335.44 | 79.56 | 155703 | 80085 | 77564 | 733 | - |
| P37057_1027 | Clogging new macadam 2021.12.14 | Macadam | 2021-12-14 | 0.093 | 322.86 | 78.71 | 151282 | 75822 | 68482 | 1777 | - |
| P37057_1028 | Macadam 16/12-20 PBS - 1 | Macadam | 2020-12-16 | 0.119 | 294.17 | 78.97 | 136536 | 69599 | 61456 | 1291 | - |
| P37057_1029 | Macadam 16/12-20 PBS - 2 | Macadam | 2020-12-16 | 0.075 | 294.2 | 79.56 | 135921 | 69958 | 63741 | 1212 | - |
| P37057_1030 | Clogging Macadam End A1-1 2022.12.06 | Biofilm_SS1 | 2022-12-06 | 0.681 | 317.81 | 79.77 | 148630 | 76297 | 50332 | 813 | - |
| P37057_1031 | Clogging Macadam End A1-2 2022.12.06 | Biofilm_SS1 | 2022-12-06 | 0.062 | 324.68 | 79.61 | 149947 | 77400 | 68173 | 1041 | - |
| P37057_1032 | Clogging Macadam End A1-3 2022.12.06 | Biofilm_SS1 | 2022-12-06 | 0.303 | 308.25 | 79.93 | 144144 | 74478 | 45203 | 775 | - |
| P37057_1033 | Clogging Macadam End B1-8 2022.12.06 | Biofilm_Tef1 | 2022-12-06 | 0.429 | 424.76 | 80.41 | 198381 | 103254 | 58009 | 814 | - |
| P37057_1034 | Clogging Macadam End B1-2 2022.12.06 | Biofilm_Tef1 | 2022-12-06 | 0.248 | 315.38 | 80.13 | 147164 | 76368 | 57404 | 897 | - |
| P37057_1035 | Clogging Macadam End B1-3 2022.12.06 | Biofilm_Tef1 | 2022-12-06 | 0.302 | 293.35 | 79.36 | 136510 | 69867 | 56146 | 948 | - |
| P37057_1036 | Clogging container 6/12-22 DNA A1 | Outlet_SS1 | 2022-12-06 | >60 | 296.78 | 80.22 | 138641 | 71269 | 37991 | 790 | 91 |
| P37057_1037 | Clogging container 6/12-22 DNA A2 | Outlet_SS2 | 2022-12-06 | >60 | 291.87 | 80.16 | 136440 | 70919 | 43399 | 1474 | 69 |
| P37057_1038 | Clogging container 6/12-22 DNA B1 | Outlet_Tef1 | 2022-12-06 | >60 | 294.02 | 80.04 | 135734 | 69142 | 37224 | 791 | 78 |
| P37057_1039 | Clogging container 6/12-22 DNA B2 | Outlet_Tef2 | 2022-12-06 | >60 | 277.54 | 79.82 | 129691 | 66922 | 36690 | 1264 | 85 |
| P37057_1040 | Control filter 6/12-22 | Control | 2022-12-06 | <0.05 | 323.66 | 79.93 | 150700 | 77812 | 73522 | 662 | - |
| P37057_1043 | Control filter 12/12-22 | Control | 2022-12-12 | <0.05 | 32.43 | 78.11 | 14948 | 7317 | 7046 | 95 | - |
| P37057_1044 | Clogging 6/12-22 New macadam - 1 | Macadam | 2022-12-12 | 0.058 | 288.53 | 78.91 | 132386 | 66714 | 63302 | 614 | - |
| P37057_1045 | Clogging 6/12-22 New macadam - 2 | Macadam | 2022-12-12 | 0.462 | 363.18 | 79.6 | 168432 | 86178 | 62433 | 1359 | - |
| P37057_1046 | Clogging 6/12-22 New macadam - 4 | Macadam | 2022-12-12 | 0.385 | 376.22 | 79.39 | 174501 | 89481 | 66356 | 1430 | - |
| P37057_1047 | Clogging container 16/2-23 DNA B1 | Outlet_Tef1 | 2023-02-16 | 51.1 | 278.7 | 80.35 | 130847 | 68184 | 33885 | 585 | 105 |
| P37057_1048 | Clogging container 23/2-23 DNA A1 | Outlet_SS1 | 2023-02-23 | >60 | 1.93 | 77.66 | 883 | 439 | 165 | 22 | 215 |
| P37057_1049 | Clogging container 15/12-23 DNA A1 | Outlet_SS1 | 2023-12-15 | >60 | 125.89 | 79.99 | 56792 | 29400 | 21607 | 1032 | 71 |
| P37057_1050 | Clogging container 15/12-23 DNA A2 | Outlet_SS2 | 2023-12-15 | >60 | 215.75 | 79.7 | 96120 | 49869 | 24956 | 854 | 147 |
| P37057_1051 | Clogging container 15/12-23 DNA B1 | Outlet_Tef1 | 2023-12-15 | >60 | 305.72 | 79.43 | 136060 | 70472 | 44662 | 1485 | 107 |
| P37057_1052 | Clogging container 15/12-23 DNA B2 | Outlet_Tef2 | 2023-12-15 | >60 | 334.68 | 79.57 | 147479 | 76369 | 42631 | 1547 | 97 |
| P37057_1053 | Control filter 15/12-23 | Control | 2023-12-15 | <0.05 | 58.94 | 81.14 | 26192 | 13606 | 10836 | 239 | - |
| P37057_1054 | Clogging container 10/1-25 DNA A1 | Outlet_SS1 | 2025-10-01 | >60 | 329.8 | 79.87 | 146047 | 75662 | 41001 | 1571 | 96 |
| P37057_1055 | Clogging container 10/1-25 DNA A2 | Outlet_SS2 | 2025-10-01 | >60 | 329.53 | 79.57 | 146684 | 75809 | 42350 | 1388 | 116 |
| P37057_1056 | Clogging container 10/1-25 DNA B1 | Outlet_Tef1 | 2025-10-01 | >60 | 231.92 | 79.92 | 105928 | 54615 | 41539 | 1591 | 99 |
| P37057_1057 | Clogging container 10/1-25 DNA B2 | Outlet_Tef2 | 2025-10-01 | 0.17 | 363.67 | 80.18 | 162806 | 84753 | 59897 | 1954 | 90 |
| P37057_1058 | Control filter 10/1-25 | Control | 2025-10-01 | <0.05 | 33.98 | 81.12 | 15292 | 7753 | 6887 | 66 | - |
| P37057_1059 | Clogging macadam end 23/1-25 A1-5 | Biofilm_SS1 | 2025-01-23 | 0.469 | 371.36 | 79.61 | 166487 | 85884 | 58693 | 1005 | - |
| P37057_1060 | Clogging macadam end 23/1-25 A1-7 | Biofilm_SS1 | 2025-01-23 | 0.138 | 335.41 | 79.97 | 149638 | 77852 | 61242 | 1041 | - |
| P37057_1061 | Clogging macadam end 23/1-25 A2-3 | Biofilm_SS2 | 2025-01-23 | <0.05 | 386.47 | 80.04 | 174488 | 90587 | 86434 | 1224 | - |
| P37057_1062 | Clogging macadam end 23/1-25 A2-7 | Biofilm_SS2 | 2025-01-23 | <0.05 | 345.6 | 79.96 | 153007 | 79353 | 70939 | 1864 | - |
| P37057_1063 | Clogging macadam end 23/1-25 B1-6 | Biofilm_Tef1 | 2025-01-23 | 0.219 | 250.52 | 80 | 114084 | 59258 | 48484 | 916 | - |
| P37057_1064 | Clogging macadam end 23/1-25 B1-7 | Biofilm_Tef1 | 2025-01-23 | 0.245 | 324.65 | 79.45 | 147148 | 75424 | 59789 | 1057 | - |
| P37057_1065 | Clogging macadam end 23/1-25 B2-5 | Biofilm_Tef2 | 2025-01-23 | 0.148 | 352.06 | 79.24 | 157196 | 81181 | 68157 | 1805 | - |
| P37057_1066 | Clogging macadam end 23/1-25 B2-7 | Biofilm_Tef2 | 2025-01-23 | 0.055 | 272.27 | 79.68 | 121183 | 62882 | 57084 | 1882 | - |
| P37057_1067 | Clogging HA0760A 25/4-25 DNA-1 | Borehole | 2025-04-24 | >60 | 346.1 | 79.03 | 153635 | 78695 | 50853 | 1948 | - |
| P37057_1068 | Clogging HA0760A 25/4-25 DNA-2 | Borehole | 2025-04-24 | >60 | 317.02 | 79.28 | 140156 | 72135 | 37281 | 1530 | - |
| P37057_1069 | Clogging Ctrl filter 25/4-25 | Control | 2025-04-24 | <0.05 | 51.96 | 80.39 | 23432 | 11807 | 10991 | 138 | - |
| P37057_1083 | Clogging macadam end 23/1-25 B1-5 | Biofilm_Tef1 | 2025-01-23 | 0.389 | 435.97 | 80.17 | 201541 | 104771 | 73765 | 1262 | - |
| P37057_1084 | Clogging macadam end 23/1-25 A2-4 | Biofilm_SS2 | 2025-01-23 | <0.05 | 484.95 | 80.62 | 223590 | 116483 | 113026 | 1407 | - |

**Supplemental Table 2** Details of the groundwater chemistry for the borehole before and after the experiment along with the outflow from the four containers.

| **Sample** | **Month** | **Na** | **K** | **Ca** | **Mg** | **Alkalinity** | **Cl** | **SO_4_** | **SO_4__S** | **F** | **Fe** | **Mn** | **pH** | **EC** | **TOC** | **DOC** | **S^2^_HS** | **NO_2__N** | **NO_3__N** | **PO_4__P** | **Temp** |
| --- | --- | --- | --- | --- | --- | --- | --- | --- | --- | --- | --- | --- | --- | --- | --- | --- | --- | --- | --- | --- | --- |
|  |  | mg/L | mg/L | mg/L | mg/L | mg/L | mg/L | mg/L | mg/L | mg/L | mg/L | mg/L |  | mS/m | mg/L | mg/L | mg/L | mg/L | mg/L | mg/L | °C |
| Borehole | -9 | 1360 | 26.2 | 209 | 104.0 | 214 | 2370 | 267 | 94.2 | 1.6 | 2.13 | 0.72 | 7.33 | 779 | NA | NA | NA | NA | NA | NA | 10.4 |
| SS1 | 0 | 1440 | 30.4 | 206 | 114.0 | 217 | 2500 | 286 | 98.0 | 1.4 | 0.36 | 0.77 | 7.48 | 818 | 8.1 | 8.1 | BD | BD | 0.0004 | 0.0024 | 11.1 |
| Tef1 | 0 | 1420 | 30.6 | 200 | 114.0 | 217 | 2490 | 287 | 97.5 | 1.4 | 0.75 | 0.75 | 7.35 | 809 | 8.6 | 8.4 | BD | 0.0003 | 0.0019 | 0.0033 | 11.3 |
| SS2 | 0 | 1410 | 30.5 | 202 | 113.0 | 217 | 2480 | 283 | 97.1 | 1.4 | 1.17 | 0.73 | 7.38 | 813 | 8.3 | 8.3 | BD | BD | BD | 0.0023 | 11.2 |
| Tef2 | 0 | 1410 | 30.4 | 204 | 113.0 | 217 | 2490 | 283 | 97.4 | 1.3 | 0.31 | 0.76 | 7.43 | 814 | 8.4 | 8.7 | BD | 0.0003 | 0.0006 | 0.0062 | 10.9 |
| SS1 | 3 | 1410 | 30.4 | 194 | 114.0 | 217 | 2480 | 287 | 98.9 | 1.4 | 1.71 | 0.73 | 7.44 | 817 | 8.4 | 8.4 | BD | 0.0008 | 0.0007 | 0.0016 | 11.9 |
| Tef1 | 3 | 1390 | 30.2 | 193 | 113.0 | 217 | 2520 | 288 | 99.1 | 1.4 | 1.58 | 0.74 | 7.45 | 816 | 8.6 | 8.4 | BD | 0.0011 | 0.0008 | 0.0018 | 11.4 |
| SS1 | 6 | 1390 | 29.9 | 187 | 107.0 | 217 | 2340 | 270 | 98.3 | 1.6 | 1.79 | 0.70 | 7.51 | 775 | 8.8 | 8.9 | BD | 0.0008 | 0.0005 | 0.0029 | 11.9 |
| Tef1 | 6 | 1360 | 34.4 | 183 | 104.0 | 217 | 2230 | 274 | 114.0 | 1.6 | BD | 0.69 | 7.62 | 783 | 8.9 | 8.6 | BD | 0.0022 | 0.0028 | 0.0023 | 12.7 |
| SS2 | 6 | 1430 | 36.3 | 198 | 113.0 | 217 | 2350 | 269 | 104.0 | 1.6 | 1.88 | 0.75 | 7.57 | 777 | 9.0 | 9.0 | BD | 0.0036 | 0.0004 | 0.0027 | 11.7 |
| Tef2 | 6 | 1410 | 30.4 | 190 | 108.0 | 217 | 2040 | 270 | 99.2 | 1.6 | 0.53 | 0.72 | 7.65 | 781 | 9.3 | 8.6 | BD | 0.0056 | 0.0015 | 0.0017 | 12.0 |
| SS1 | 9 | 1190 | 27.3 | 179 | 102.0 | 221 | 2110 | 251 | 91.2 | 1.6 | 0.91 | 0.64 | 7.53 | 721 | 9.0 | 8.9 | BD | 0.0018 | 0.0027 | 0.0024 | 12.6 |
| Tef1 | 9 | 1210 | 27.3 | 179 | 102.0 | 219 | 2120 | 253 | 91.0 | 1.5 | 0.81 | 0.64 | 7.67 | 722 | 9.0 | 9.0 | BD | 0.0018 | 0.0024 | 0.0027 | 12.2 |
| SS1 | 12 | 1200 | 26.2 | 180 | 99.5 | 220 | 2180 | 248 | 89.2 | 1.6 | 1.44 | 0.66 | 7.62 | 733 | 8.9 | 8.9 | BD | 0.0013 | 0.0049 | 0.0029 | 11.2 |
| Tef1 | 12 | 1200 | 26.0 | 180 | 98.9 | 219 | 2200 | 249 | 89.2 | 1.6 | 1.50 | 0.64 | 7.61 | 741 | 8.8 | 8.7 | BD | 0.0010 | 0.0024 | 0.0026 | 11.2 |
| SS2 | 12 | 1200 | 26.1 | 179 | 98.9 | 219 | 2220 | 249 | 89.0 | 1.5 | 0.68 | 0.65 | 7.61 | 741 | 8.9 | 8.7 | BD | 0.0011 | 0.0017 | 0.0024 | 11.2 |
| Tef2 | 12 | 1190 | 25.9 | 180 | 98.6 | 218 | 2210 | 249 | 89.0 | 1.6 | 0.73 | 0.64 | 7.63 | 745 | 9.1 | 8.7 | BD | 0.0010 | 0.0007 | 0.0020 | 11.3 |
| SS2 | 18 | 1230 | 25.2 | 154 | 96.5 | 229 | 2000 | 234 | 85.6 | 1.6 | 1.48 | 0.63 | 7.67 | 682 | 11.6 | 11.6 | BD | 0.0020 | 0.0026 | 0.0061 | 11.3 |
| Tef2 | 18 | 1220 | 25.0 | 152 | 95.9 | 229 | 1990 | 234 | 84.6 | 1.6 | 2.00 | 0.62 | 7.62 | 683 | 11.8 | 12.0 | BD | 0.0032 | 0.0025 | 0.0067 | 11.4 |
| SS2 | 24 | 1230 | 25.7 | 165 | 89.9 | 234 | 1970 | 238 | 86.4 | 1.6 | 1.69 | 0.63 | 7.49 | 672 | 10.8 | 10.9 | BD | 0.0006 | 0.0013 | 0.0032 | NA |
| Tef2 | 24 | 1260 | 25.4 | 164 | 90.2 | 234 | 1980 | 240 | 85.7 | 1.5 | 1.69 | 0.63 | 7.45 | 676 | 10.9 | 10.9 | BD | 0.0005 | 0.0016 | 0.0028 | NA |
| SS2 | 30 | 1260 | 27.2 | 164 | 102.0 | 222 | 2140 | 250 | 88.8 | 1.5 | 1.66 | 0.63 | 7.51 | 713 | 10.2 | 10.1 | BD | 0.0010 | 0.0000 | 0.0030 | NA |
| Tef2 | 30 | 1210 | 27.1 | 166 | 102.0 | 222 | 2140 | 252 | 89.0 | 1.5 | 1.61 | 0.63 | 7.56 | 714 | 10.2 | 9.9 | BD | 0.0007 | BD | 0.0037 | NA |
| SS2 | 36 | 1240 | 25.7 | 160 | 93.1 | 225 | 2090 | 240 | 82.2 | 1.5 | 1.73 | 0.62 | 7.64 | 696 | 11.0 | 11.0 | BD | 0.0010 | 0.0020 | 0.0180 | NA |
| Tef2 | 36 | 1230 | 25.2 | 161 | 93.2 | 225 | 2100 | 250 | 82.7 | 1.5 | 1.70 | 0.62 | 7.53 | 696 | 11.0 | 11.0 | BD | 0.0010 | 0.0010 | 0.0200 | NA |
| SS1 | 37 | 1230 | 26.8 | 167 | 94.5 | 223 | 2190 | 260 | 86.1 | 1.6 | 1.47 | 0.64 | 7.42 | 724 | 9.8 | 9.5 | BD | 0.0049 | 0.0006 | 0.0012 | NA |
| Tef1 | 37 | 1230 | 26.8 | 167 | 93.7 | 222 | 2180 | 240 | 85.3 | 1.6 | 1.59 | 0.64 | 7.41 | 719 | 9.6 | 9.5 | BD | 0.0004 | 0.0004 | 0.0013 | NA |
| SS2 | 42 | 1230 | 25.9 | 167 | 95.9 | 221 | 2110 | 250 | 86.0 | 1.6 | 1.76 | 0.63 | 7.51 | 698 | 10.0 | 10.0 | BD | 0.0006 | 0.0010 | 0.0024 | NA |
| Tef2 | 42 | 1240 | 26.1 | 168 | 97.6 | 221 | 2120 | 250 | 86.8 | 1.6 | 1.78 | 0.64 | 7.58 | 703 | 10.0 | 9.8 | BD | 0.0005 | 0.0011 | 0.0024 | NA |
| SS2 | 49 | 1240 | 26.8 | 167 | 94.6 | 223 | 2190 | 260 | 86.6 | 1.5 | 1.46 | 0.65 | 7.38 | 721 | 9.4 | 9.2 | BD | 0.0004 | 0.0028 | 0.0029 | NA |
| Tef2 | 49 | 1260 | 27.3 | 170 | 96.8 | 220 | 2210 | 250 | 88.5 | 1.5 | 1.58 | 0.66 | 7.58 | 722 | 9.4 | 9.4 | BD | 0.0005 | 0.0009 | 0.0038 | NA |
| Borehole | 49 | 1210 | 26.7 | 166 | 93.3 | 222 | 2190 | 260 | 85.4 | 1.5 | 1.14 | 0.63 | 7.42 | 714 | 10.0 | 9.8 | 0.03 | 0.0004 | 0.0003 | 0.0013 | NA |

NA, not available

BD, below detection

**Supplemental Table 3** List of pathways, genes, KO identifiers, and description searched for in the PiCRUSt2 analysis.

| **Pathway** | **KO identifier** | **Gene ID** | **Description** |
| --- | --- | --- | --- |
| Hydrogen oxidation, anaerobic, NADP | ElP_32890 | *hndA* | NADH:ubiquinone reductase (H+-translocating) |
| Hydrogen oxidation, anaerobic, NADP | K17992 | *hndB* | NADP-reducing hydrogenase subunit HndB |
| Hydrogen oxidation, anaerobic, NADP | K18331 | *hndC* | NADP-reducing hydrogenase subunit HndC |
| Hydrogen oxidation, anaerobic, NADP | K18332 | *hndD* | NADP-reducing hydrogenase subunit HndD |
| Sulfide oxidation | K17230 | *fccA* | Cytochrome subunit of sulfide dehydrogenase (Flavocytochrome c cytochrome subunit) |
| Sulfide oxidation | K17229 | *fccB* | Sulfide dehydrogenase [flavocytochrome c] flavoprotein chain (EC 1.8.2.3) (Flavocytochrome c flavoprotein subunit) |
| S oxidation | K16952 | *sor* | Sulfur oxygenase/reductase (EC 1.13.11.55) (Sulfur oxygenase reductase) SOR |
| S oxidation | K03388 | *hdrA* | H(2):CoB-CoM heterodisulfide.ferredoxin reductase subunit A (EC 1.8.98.5) (CoB--CoM heterodisulfide reductase iron-sulfur subunit A) |
| S oxidation | K03389 | *hdrB* | H(2):CoB-CoM heterodisulfide.ferredoxin reductase subunit B (EC 1.8.98.5) (CoB--CoM heterodisulfide reductase subunit B) |
| Thiosulfate oxidation | K17226 | *soxY* | Sulfur-oxidizing protein SoxY |
| Thiosulfate oxidation | K17223 | *soxX* | L-cysteine S-thiosulfotransferase |
| Thiosulfate oxidation | K17227 | *soxZ* | Sulfur-oxidizing protein SoxZ |
| Thiosulfate oxidation | K17222 | *soxA* | L-cysteine S-thiosulfotransferase |
| Thiosulfate oxidation | K17224 | *soxB* | S-sulfosulfanyl-L-cysteine sulfohydrolase |
| Thiosulfate oxidation | K17225 | *soxC* | Sulfane dehydrogenase subunit SoxC |
| Thiosulfate oxidation | K16936 | *doxA* | Thiosulfate dehydrogenase (quinone) small subunit |
| Thiosulfate oxidation | K16937 | *doxD* | Thiosulfate dehydrogenase (quinone) large subunit |
| Thiosulfate oxidation | K05908 | *doxDA* | Thiosulfate dehydrogenase (quinone) [EC:1.8.5.2] |
| Thiosulfate oxidation | K19713 | *tsdA* | Thiosulfate dehydrogenase |
| Tetrathionate hydrolase | K27925 | *tetH* | Tetrathionate hydrolase (4THase) (TTH) (EC 3.12.1.-) |
| Methane oxidation, methanotroph, methane => formaldehyde | K14028 | *mdh1*, *mxaF* | Methanol dehydrogenase (cytochrome c) subunit 1 |
| Methane oxidation, methanotroph, methane => formaldehyde | K14029 | *mdh2*, *mxaI* | Methanol dehydrogenase (cytochrome c) subunit 2 |
| Methane oxidation, methanotroph, methane => formaldehyde | K16160 | *mmoB* | Methane monooxygenase regulatory protein B |
| Methane oxidation, methanotroph, methane => formaldehyde | K16161 | *mmoC* | Methane monooxygenase component C |
| Methane oxidation, methanotroph, methane => formaldehyde | K16162 | *mmoD* | Methane monooxygenase component D |
| Methane oxidation, methanotroph, methane => formaldehyde | K16157 | *mmoX* | Methane monooxygenase component A alpha chain |
| Methane oxidation, methanotroph, methane => formaldehyde | K16158 | *mmoY* | Methane monooxygenase component A beta chain |
| Methane oxidation, methanotroph, methane => formaldehyde | K16159 | *mmoZ* | Methane monooxygenase component A gamma chain |
| Methane oxidation, methanotroph, methane => formaldehyde | K10944 | *pmoA*, *amoA* | methane/ammonia monooxygenase subunit A |
| Methane oxidation, methanotroph, methane => formaldehyde | K10945 | *pmoB*, *amoB* | methane/ammonia monooxygenase subunit B |
| Methane oxidation, methanotroph, methane => formaldehyde | K10946 | *pmoC*, *amoC* | methane/ammonia monooxygenase subunit C |
| Dissimilatory nitrate reduction | K02567 | *napA* | Nitrate reductase (cytochrome) |
| Dissimilatory nitrate reduction | K02568 | *napB* | Nitrate reductase (cytochrome), electron transfer subunit |
| Dissimilatory nitrate reduction | K00370 | *narG, narZ, nxrA* | Nitrate reductase /nitrite oxidoreductase, alpha subunit |
| Dissimilatory nitrate reduction | K00371 | *narH*, *narY*, *nxrB* | Nitrate reductase / nitrite oxidoreductase, beta subunit |
| Dissimilatory nitrate reduction | K00374 | *narI*, *narV* | Nitrate reductase gamma subunit |
| Dissimilatory nitrate reduction | K00373 | *narJ*, *narW* | Nitrate reductase molybdenum cofactor assembly chaperone |
| Dissimilatory nitrate reduction, DNRA or ANR | K00362 | *nirB* | Nitrite reductase (NADH) large subunit |
| Dissimilatory nitrate reduction, DNRA or ANR | K00363 | *nirD* | nitrite reductase (NADH) small subunit |
| Dissimilatory nitrate reduction, DNRA | K03385 | *nrfA* | Nitrite reductase (cytochrome c-552) |
| Dissimilatory nitrate reduction, DNRA | K15876 | *nrfH* | Cytochrome c nitrite reductase small subunit |
| Denitrification | K15877 | *CYP55* | Fungal nitric oxide reductase |
| Denitrification | K00368 | *nirK* | Nitrite reductase (NO-forming) |
| Denitrification | K15864 | *nirS* | Nitrite reductase (NO-forming) / hydroxylamine reductase |
| Denitrification | K04561 | *norB* | Nitric oxide reductase subunit B |
| Denitrification | K02305 | *norC* | Nitric oxide reductase subunit C |
| Denitrification | K00376 | *nosZ* | Nitrous-oxide reductase |
| Dissimilatory sulfate reduction, sulfate => H2S | K00394 | *aprA* | Adenylylsulfate reductase, subunit A |
| Dissimilatory sulfate reduction, sulfate => H2S | K00395 | *aprB* | Adenylylsulfate reductase, subunit B |
| Dissimilatory sulfate reduction, sulfate => H2S | K11180 | *dsrA* | Dissimilatory sulfite reductase alpha subunit |
| Dissimilatory sulfate reduction, sulfate => H2S | K11181 | *dsrB* | Dissimilatory sulfite reductase beta subunit |
| Dissimilatory sulfate reduction, sulfate => H2S | K27188 | *dsrK* | [DsrC]-trisulfide reductase subunit K |
| Dissimilatory sulfate reduction, sulfate => H2S | K00958 | *sat*, *met3* | Sulfate adenylyltransferase |
| Sulfite reduction | K16950 | *asrA* | Anaerobic sulfite reductase subunit A |
| Sulfite reduction | K16951 | *asrB* | Anaerobic sulfite reductase subunit B |
| Sulfite reduction | K00385 | *asrC* | Anaerobic sulfite reductase subunit C |
| Tetrathionate reduction | K08357 | *ttrA* | Tetrathionate reductase subunit A |
| Tetrathionate reduction | K08358 | *ttrB* | Tetrathionate reductase subunit B |
| Tetrathionate reduction | K08359 | *ttrC* | Tetrathionate reductase subunit C |
| Thiosulfate reduction | K08352 | *phsA*, *psrA* | Thiosulfate reductase / polysulfide reductase chain A |
| Thiosulfate reduction | K08353 | *phsB* | Thiosulfate reductase electron transport protein |
| Thiosulfate reduction | K08354 | *phsC* | Thiosulfate reductase cytochrome b subunit |
| Arsenate reduction | EKN56_13105 | *arrA* | Arsenate reductase (donor) |
| Arsenate reduction | EKN56_13100 | *arrB* | Arsenate reductase (donor) iron-sulfur subunit |
| Selenate reduction | CAB53372 | *serA* | Selenate/chlorate reductase subunit alpha [EC:1.97.1.9 1.97.1.1] |
| Selenate reduction | CAB53373 | *serB* | Selenate/chlorate reductase subunit beta [EC:1.97.1.9 1.97.1.1] |
| Selenate reduction | CAB53375 | *serC* | Selenate/chlorate reductase subunit gamma [EC:1.97.1.9 1.97.1.1] |
| Methanogenesis, all | K22480 | *hdrA1* | Heterodisulfide reductase subunit A1 |
| Methanogenesis, all | K03388 | *hdrA2* | Heterodisulfide reductase subunit A2 |
| Methanogenesis, all | K22481 | *hdrB1* | Heterodisulfide reductase subunit B1 |
| Methanogenesis, all | K03389 | *hdrB2* | Heterodisulfide reductase subunit B2 |
| Methanogenesis, all | K22482 | *hdrC1* | Heterodisulfide reductase subunit C1 |
| Methanogenesis, all | K03390 | *hdrC2* | Heterodisulfide reductase subunit C2 |
| Methanogenesis, all | K00399 | *mcrA* | Methyl-coenzyme M reductase alpha subunit |
| Methanogenesis, all | K00401 | *mcrB* | Methyl-coenzyme M reductase beta subunit |
| Methanogenesis, all | K00402 | *mcrG* | Methyl-coenzyme M reductase gamma subunit |
| Methanogenesis, CO2 => methane | K00672 | *ftr* | Formylmethanofuran--tetrahydromethanopterin Nformyltransferase |
| Methanogenesis, CO2 => methane | K00200 | *fwdA*, *fmdA* | Formylmethanofuran dehydrogenase subunit A |
| Methanogenesis, CO2 => methane | K00201 | *fwdB*, *fmdB* | Formylmethanofuran dehydrogenase subunit B |
| Methanogenesis, CO2 => methane | K00202 | *fwdC*, *fmdC* | Formylmethanofuran dehydrogenase subunit C |
| Methanogenesis, CO2 => methane | K00203 | *fwdD*, *fmdD* | Formylmethanofuran dehydrogenase subunit D |
| Methanogenesis, CO2 => methane | K11261 | *fwdE*, *fmdE* | Formylmethanofuran dehydrogenase subunit E |
| Methanogenesis, CO2 => methane | K00205 | *fwdF*, *fmdF* | 4Fe-4S ferredoxin |
| Methanogenesis, CO2 => methane | K11260 | *fwdG* | 4Fe-4S ferredoxin |
| Methanogenesis, CO2 => methane | K00204 | *fwdH* | 4Fe-4S ferredoxin |
| Methanogenesis, CO2 => methane | K13942 | *hmd* | 5,10-methenyltetrahydromethanopterin hydrogenase |
| Methanogenesis, CO2 => methane | K01499 | *mch* | Methenyltetrahydromethanopterin cyclohydrolase |
| Methanogenesis, CO2 => methane | K00320 | *mer* | 5,10-methylenetetrahydromethanopterin reductase |
| Methanogenesis, CO2 => methane | K00319 | *mtd* | Methylenetetrahydromethanopterin dehydrogenase |
| Methanogenesis, CO2 => methane or acetate => methane | K00577 | *mtrA* | Tetrahydromethanopterin S-methyltransferase subunit A |
| Methanogenesis, CO2 => methane or acetate => methane | K00578 | *mtrB* | Tetrahydromethanopterin S-methyltransferase subunit B |
| Methanogenesis, CO2 => methane or acetate => methane | K00579 | *mtrC* | Tetrahydromethanopterin S-methyltransferase subunit C |
| Methanogenesis, CO2 => methane or acetate => methane | K00580 | *mtrD* | Tetrahydromethanopterin S-methyltransferase subunit D |
| Methanogenesis, CO2 => methane or acetate => methane | K00581 | *mtrE* | Tetrahydromethanopterin S-methyltransferase subunit E |
| Methanogenesis, CO2 => methane or acetate => methane | K00582 | *mtrF* | Tetrahydromethanopterin S-methyltransferase subunit F |
| Methanogenesis, CO2 => methane or acetate => methane | K00583 | *mtrG* | Tetrahydromethanopterin S-methyltransferase subunit G |
| Methanogenesis, CO2 => methane or acetate => methane | K00584 | *mtrH* | Tetrahydromethanopterin S-methyltransferase subunit H |
| Methanogenesis, methanol => methane | K14080 | *mtaA* | [Methyl-Co(III) methanol/glycine betaine-specific corrinoid protein]:coenzyme M methyltransferase |
| Methanogenesis, methanol => methane | K04480 | *mtaB* | Methanol---5-hydroxybenzimidazolylcobamide Comethyltransferase |
| Methanogenesis, methylamine/dimethylamine/trimethylamine => methane | K14082 | *mtbA* | [Methyl-Co(III) methylamine-specific corrinoid protein]:coenzyme M methyltransferase |
| Methanogenesis, methylamine/dimethylamine/trimethylamine => methane | K16178 | *mtbB* | Dimethylamine---corrinoid protein Comethyltransferase |
| Methanogenesis, methylamine/dimethylamine/trimethylamine => methane | K16176 | *mtmB* | Methylamine---corrinoid protein Co-methyltransferase |
| Methanogenesis, methylamine/dimethylamine/trimethylamine => methane | K14083 | *mttB* | Trimethylamine---corrinoid protein Comethyltransferase |
| Methanogenesis from glycine betaine | K25220 | *mtgB* | Glycine betaine---corrinoid protein Co-methyltransferas |
| Carbon fixation, 3HP/4HB | K14468 | *mcr* | Malonyl-CoA reductase / 3-hydroxypropionate dehydrogenase (NADP+) |
| Carbon fixation, 3HP/4HB | K14449 | *mch*, *mcd* | 2-methylfumaryl-CoA hydratase |
| Carbon fixation, CBB cycle (Rubisco) | K01601 | *rbcL*, *cbbL* | Ribulose-bisphosphate carboxylase large chain |
| Carbon fixation, CBB cycle (Rubisco) | K01602 | *rbcS*, *cbbS* | Ribulose-bisphosphate carboxylase small chain |
| Carbon fixation, CBB cycle (Rubisco) | K00855 | *prk* | Phosphoribulokinase |
| Carbon fixation, reverse TCA cycle | K15230 | *aclA* | ATP-citrate lyase alpha-subunit |
| Carbon fixation, reverse TCA cycle | K15231 | *aclB* | ATP-citrate lyase beta-subunit |
| Carbon fixation, reverse TCA cycle | K15232 | *ccsA* | Citryl-CoA synthetase large subunit |
| Carbon fixation, reverse TCA cycle | K15233 | *ccsB* | Citryl-CoA synthetase small subunit |
| Carbon fixation, reverse TCA cycle | K15234 | *ccl* | Citryl-CoA lyase |
| Carbon fixation, reverse TCA cycle | K00174 | *korA*, *oorA*, *oforA* | 2-oxoglutarate/2-oxoacid ferredoxin oxidoreductase subunit alpha |
| Carbon fixation, reverse TCA cycle | PAB0344 | *korB* | 2-oxoacid oxidoreductase (ferredoxin) |
| Carbon fixation, reverse TCA cycle | K00244 | *frdA* | Fumarate reductase flavoprotein subunit (EC 1.3.5.4) |
| Carbon fixation, reverse TCA cycle | K00245 | *frdB* | Fumarate reductase iron-sulfur subunit (EC 1.3.5.1) |
| Carbon fixation, Wood−Ljungdahl | K00198 | *cooS*, *acsA* | Anaerobic carbon-monoxide dehydrogenase catalytic subunit |
| Carbon fixation, Wood−Ljungdahl | K14138 | *acsB* | Acetyl-CoA synthase |
| Carbon fixation, Wood−Ljungdahl | K00197 | *cdhE*, *acsC* | Acetyl-CoA decarbonylase/synthase, CODH/ACS complex subunit gamma |
| Carbon fixation, Wood−Ljungdahl | K01500 | *fchA* | Methenyltetrahydrofolate cyclohydrolase |
| Carbon fixation, Wood−Ljungdahl | K00194 | *cdhD*, *acsD* | Acetyl-CoA decarbonylase/synthase, CODH/ACS complex subunit delta |
| Nitrogen fixation | K02586 | *nifD* | Nitrogenase molybdenum-iron protein alpha chain |
| Nitrogen fixation | K02591 | *nifK* | Nitrogenase molybdenum-iron protein beta chain |
| Nitrogen fixation | K02588 | *nifH* | Nitrogenase iron protein NifH |

**Supplemental Table 4** Statistical support for comparison of the 16S rRNA gene-based beta diversity based upon ANOVA, PERMANOVA, and MANOVA testing of the populations for all samples.

ANOVA dispersion

Response: Distances

Df Sum Sq Mean Sq F value Pr(>F)

Groups 5 0.13599 0.027199 1.3436 0.2605

Residuals 53 1.07290 0.020243

PERMANOVA:

Permutation test for adonis under reduced model

Permutation: free

Number of permutations: 9999

adonis2(formula = bray curtis dissimilarity * Incubation time in yrs * sample type, data = samples_nmds_perm)

Df SumOfSqs R2 F Pr(>F)

Model 18 12.4243 0.5575 2.7977 1e-04 ***

Residual 40 9.8614 0.4425

Total 58 22.2857 1.00000

---

Signif. codes: 0 ‘***’ 0.001 ‘**’ 0.01 ‘*’ 0.05 ‘.’ 0.1 ‘ ’ 1

**16S rRNA gene based pairwise comparisons of ASVs using MANOVAs on a Bray Curtis dissimilarity matrix for combined SS and Tef samples with 9999 permutations.** The replicate numbers for the different samples are given in the table. Significance defined as *p*<0.05 (in bold) with FDR adjustment.

|  | 1.Biofilm  (*n*=11) | 2.Biofilm  (*n*=5) | 4.Biofilm  (*n*=5) | 0.Borehole  (*n*=4) | 4.Borehole  (*n*=2) | 0.Macadam  (*n*=8) | 0.Outlet  (*n*=8) | 1.Outlet  (*n*=8) | 2.Outlet  (*n*=4) | 3.Outlet  (*n*=2) |
| --- | --- | --- | --- | --- | --- | --- | --- | --- | --- | --- |
| 2.Biofilm (*n*=5) | **0.0031** | - | - | - | - | - | - | - | - | - |
| 4.Biofilm (*n*=5) | **0.0031** | **0.0201** | - | - | - | - | - | - | - | - |
| 0.Borehole (*n*=4) | **0.0038** | **0.0201** | **0.0196** | - | - | - | - | - | - | - |
| 4.Borehole (*n*=2) | **0.0244** | 0.0595 | 0.0595 | 0.4074 | - | - | - | - | - | - |
| 0.Macadam (*n*=8) | **0.0032** | **0.0032** | **0.0038** | **0.0071** | **0.0418** | - | - | - | - | - |
| 0.Outlet (*n*=8) | **0.0018** | **0.0038** | **0.0032** | **0.046** | 0.0595 | **0.0022** | - | - | - | - |
| 1.Outlet (*n*=8) | **0.0018** | **0.0032** | **0.0034** | **0.0168** | **0.04** | **0.0022** | **0.0018** | - | - | - |
| 2.Outlet (*n*=4) | **0.0032** | **0.0209** | **0.0202** | **0.0432** | 0.156 | **0.0086** | **0.0168** | **0.0416** | - | - |
| 3.Outlet (*n*=2) | **0.0244** | 0.0595 | 0.0595 | 0.4074 | 0.3595 | **0.0416** | 0.1345 | 0.0595 | 0.3595 | - |
| 4.Outllet (*n*=2) | **0.0244** | 0.0595 | 0.0595 | 0.4074 | 0.3595 | **0.0416** | 0.0808 | 0.0595 | 0.3595 | 0.6667 |

Abbreviations: The codes are the incubation time of 0, 1, 2, 3, and 4 years plus sample type of borehole, biofilm, macadam, and outlet.

**Supplemental Table 5** 16S rRNA gene based relative abundances at the family and genus levels.

|  | **Borehole** | | | | **Biofilm** | | | | | | **Outlet** | | | | | | | | | |
| --- | --- | --- | --- | --- | --- | --- | --- | --- | --- | --- | --- | --- | --- | --- | --- | --- | --- | --- | --- | --- |
| **Incubation time (years)** | **0** | | **4** | | **1** | | **2** | | **4** | | **0** | | **1** | | **2** | | **3** | | **4** | |
|  | **Mean** | **SD** | **Mean** | **SD** | **Mean** | **SD** | **Mean** | **SD** | **Mean** | **SD** | **Mean** | **SD** | **Mean** | **SD** | **Mean** | **SD** | **Mean** | **SD** | **Mean** | **SD** |
| Other | 53.7 | 4.7 | 59.3 | 1.9 | 65.2 | 16.6 | 64.2 | 3.6 | 83.7 | 2.9 | 31.0 | 19.3 | 65.3 | 10.1 | 61.4 | 5.8 | 48.7 | 17.7 | 55.4 | 16.7 |
| Acidaminobacteraceae | 4.3 | 8.7 | NI | NI | 4.3 | 3.8 | 4.8 | 1.3 | 0.2 | 0.2 | 0.0 | 0.0 | 0.1 | 0.1 | 0.0 | 0.0 | NI | NI | NI | NI |
| BM004 | 0.7 | 1.2 | 0.2 | 0.2 | 0.1 | 0.3 | 1.1 | 0.4 | 0.8 | 0.8 | 4.9 | 9.2 | 2.2 | 1.7 | 1.3 | 0.6 | 0.4 | 0.1 | 1.9 | 2.2 |
| Burkholderiaceae | 0.0 | 0.0 | 0.0 | 0.0 | 2.7 | 2.9 | 4.9 | 3.1 | 2.5 | 1.0 | 4.7 | 5.6 | 0.1 | 0.0 | 0.1 | 0.1 | 0.0 | 0.0 | 0.0 | 0.0 |
| Clostridiaceae | 0.0 | 0.0 | NI | NI | 5.2 | 4.2 | 0.6 | 0.2 | 0.7 | 0.5 | 7.2 | 7.9 | 0.2 | 0.1 | 0.0 | 0.0 | NI | NI | NI | NI |
| Desulfitobacteriaceae | 0.0 | 0.0 | 0.1 | 0.0 | 5.6 | 6.4 | 0.0 | 0.0 | 0.0 | 0.0 | 0.1 | 0.4 | 0.3 | 0.5 | 0.0 | 0.0 | 0.0 | 0.0 | 0.0 | 0.0 |
| Desulfobacteraceae | 0.0 | 0.0 | NI | NI | 1.7 | 1.8 | 2.5 | 1.6 | 0.7 | 0.3 | 0.3 | 0.5 | 4.4 | 4.2 | 0.8 | 0.5 | 0.4 | 0.3 | 0.3 | 0.3 |
| Desulfocapsaceae | 1.9 | 3.7 | NI | NI | 3.1 | 3.0 | 5.8 | 1.8 | 1.8 | 0.8 | 0.6 | 1.1 | 0.2 | 0.1 | 0.0 | 0.0 | 0.0 | 0.0 | NI | NI |
| Enterobacteriaceae | 0.0 | 0.1 | NI | NI | 0.1 | 0.3 | 0.0 | 0.0 | 0.0 | 0.0 | 9.8 | 11.9 | NI | NI | NI | NI | NI | NI | NI | NI |
| GW2011-AR1 | 4.2 | 3.6 | 5.7 | 0.7 | 0.0 | 0.0 | 0.8 | 0.1 | 0.2 | 0.1 | 1.1 | 1.0 | 1.6 | 1.4 | 3.2 | 1.0 | 2.1 | 0.8 | 2.4 | 0.1 |
| GWC2-42-12 | 4.1 | 2.7 | 5.3 | 0.8 | 0.1 | 0.1 | 0.0 | 0.0 | 0.0 | 0.0 | 1.3 | 1.2 | 2.3 | 1.1 | 4.7 | 1.1 | 4.9 | 0.3 | 3.9 | 1.7 |
| Gorgyraeaceae | 4.1 | 2.9 | 4.2 | 0.3 | 0.0 | 0.0 | 0.0 | 0.0 | 0.0 | 0.0 | 0.3 | 0.4 | 1.0 | 0.9 | 2.8 | 1.3 | 2.6 | 0.6 | 2.7 | 0.7 |
| Holophagaceae | 4.1 | 8.3 | NI | NI | 1.4 | 1.5 | 4.5 | 0.8 | 0.0 | 0.0 | 0.0 | 0.1 | 0.0 | 0.0 | 0.0 | 0.0 | 0.0 | 0.0 | NI | NI |
| Pluralincolimonadaceae | 3.5 | 2.4 | 3.2 | 0.1 | 0.0 | 0.1 | 0.0 | 0.0 | 0.0 | 0.0 | 0.9 | 1.3 | 1.2 | 0.9 | 2.5 | 1.0 | 2.6 | 0.4 | 2.3 | 1.3 |
| Profunditerraquicolaceae | 11.3 | 7.6 | 14.3 | 0.2 | 0.0 | 0.1 | 0.1 | 0.0 | 0.8 | 0.5 | 2.2 | 2.2 | 4.7 | 4.0 | 10.3 | 4.8 | 9.7 | 2.7 | 9.9 | 3.5 |
| Prolixibacteraceae | 1.1 | 2.1 | NI | NI | 5.6 | 6.2 | 2.6 | 0.4 | 0.2 | 0.1 | 0.0 | 0.0 | 0.2 | 0.1 | 0.0 | 0.0 | 0.0 | 0.0 | 0.0 | 0.0 |
| Pseudomonadaceae | 0.1 | 0.2 | 0.8 | 1.1 | 0.2 | 0.3 | 0.0 | 0.0 | 0.2 | 0.1 | 29.6 | 31.9 | 0.2 | 0.3 | 0.1 | 0.1 | 0.0 | 0.0 | NI | NI |
| Rhodocyclaceae | 0.1 | 0.1 | NI | NI | 0.3 | 0.4 | 0.2 | 0.1 | 1.7 | 1.0 | 4.6 | 3.6 | 11.3 | 13.1 | 7.2 | 6.3 | 22.4 | 22.6 | 15.8 | 11.5 |
| UBA2206 | 1.2 | 0.9 | 1.1 | 0.2 | 0.2 | 0.2 | 0.3 | 0.1 | 0.6 | 0.4 | 0.5 | 0.4 | 3.2 | 1.7 | 1.5 | 0.3 | 2.2 | 0.4 | 1.8 | 0.4 |
| UBA5619 | 5.7 | 2.0 | 5.8 | 1.0 | 0.2 | 0.1 | 1.6 | 0.2 | 4.3 | 1.6 | 0.8 | 0.8 | 1.8 | 0.6 | 3.9 | 0.6 | 3.9 | 0.0 | 3.4 | 0.9 |
| Sphingomonadaceae | NI | NI | NI | NI | 4.0 | 2.7 | 6.0 | 0.7 | 1.2 | 0.4 | 0.1 | 0.2 | 0.0 | 0.0 | 0.0 | 0.0 | NI | NI | NI | NI |

NI, not identified

|  | **Borehole** | | | | **Biofilm** | | | | | | **Outlet** | | | | | | | | | |
| --- | --- | --- | --- | --- | --- | --- | --- | --- | --- | --- | --- | --- | --- | --- | --- | --- | --- | --- | --- | --- |
| **Incubation time (years)** | **0** | | **4** | | **1** | | **2** | | **4** | | **0** | | **1** | | **2** | | **3** | | **4** | |
|  | **Mean** | **SD** | **Mean** | **SD** | **Mean** | **SD** | **Mean** | **SD** | **Mean** | **SD** | **Mean** | **SD** | **Mean** | **SD** | **Mean** | **SD** | **Mean** | **SD** | **Mean** | **SD** |
| Other | 67.7 | 9.3 | 73.2 | 2.6 | 71.5 | 14.6 | 76.9 | 2.3 | 89.1 | 3.0 | 52.3 | 21.4 | 79.2 | 4.7 | 74.1 | 3.4 | 79.0 | 2.5 | 80.6 | 4.6 |
| *Aquipluma* | 1.1 | 2.1 | NI | NI | 5.3 | 5.9 | 1.1 | 0.7 | 0.1 | 0.1 | NI | NI | 0.0 | 0.0 | NI | NI | NI | NI | NI | NI |
| *C7867-001* | 0.5 | 0.4 | 1.0 | 0.1 | 0.0 | 0.0 | 0.3 | 0.1 | 0.7 | 0.7 | 0.5 | 0.9 | 1.6 | 1.2 | 2.0 | 0.2 | 1.5 | 0.1 | 1.4 | 0.5 |
| *Clostridium_AD* | 0.0 | 0.0 | NI | NI | 1.3 | 1.0 | 0.0 | 0.0 | 0.1 | 0.0 | 4.5 | 5.3 | 0.2 | 0.1 | 0.0 | 0.0 | NI | NI | NI | NI |
| *DYTH01* | 5.7 | 1.9 | 5.7 | 1.0 | 0.2 | 0.1 | 1.6 | 0.2 | 4.3 | 1.6 | 0.8 | 0.8 | 1.8 | 0.6 | 3.9 | 0.6 | 3.9 | 0.0 | 3.4 | 0.9 |
| *Desulfobacula* | 0.0 | 0.0 | NI | NI | 1.6 | 1.7 | 2.2 | 1.3 | 0.5 | 0.1 | 0.2 | 0.5 | 4.2 | 4.0 | 0.8 | 0.5 | 0.3 | 0.2 | 0.3 | 0.2 |
| *Desulforhopalus* | 0.0 | 0.0 | NI | NI | 2.4 | 2.7 | 1.3 | 0.3 | 1.2 | 0.9 | 0.2 | 0.6 | 0.1 | 0.1 | 0.0 | 0.0 | 0.0 | 0.0 | NI | NI |
| *Desulfosporosinus* | 0.0 | 0.0 | 0.1 | 0.0 | 5.6 | 6.4 | 0.0 | 0.0 | 0.0 | 0.0 | 0.1 | 0.4 | 0.3 | 0.5 | 0.0 | 0.0 | 0.0 | 0.0 | 0.0 | 0.0 |
| *Fusibacter_C* | 4.3 | 8.7 | NI | NI | 4.1 | 3.7 | 4.7 | 1.4 | 0.2 | 0.2 | 0.0 | 0.0 | 0.0 | 0.0 | NI | NI | NI | NI | NI | NI |
| *JACPHD01* | 4.0 | 2.7 | 5.3 | 0.8 | 0.1 | 0.1 | 0.0 | 0.0 | 0.0 | 0.0 | 1.3 | 1.2 | 2.3 | 1.1 | 4.7 | 1.1 | 4.8 | 0.3 | 3.9 | 1.7 |
| *JAKITW01* | 0.7 | 1.2 | 0.2 | 0.2 | 0.1 | 0.3 | 1.1 | 0.4 | 0.8 | 0.8 | 4.9 | 9.2 | 2.2 | 1.7 | 1.3 | 0.6 | 0.4 | 0.1 | 1.9 | 2.2 |
| *MWBB01* | 4.1 | 8.3 | NI | NI | 1.4 | 1.5 | 4.4 | 0.8 | 0.0 | 0.0 | 0.0 | 0.1 | 0.0 | 0.0 | NI | NI | NI | NI | NI | NI |
| *Pluralincolimonas* | 3.2 | 2.2 | 2.9 | 0.1 | 0.0 | 0.1 | 0.0 | 0.0 | 0.0 | 0.0 | 0.9 | 1.3 | 1.1 | 0.9 | 2.3 | 1.0 | 2.5 | 0.3 | 2.1 | 1.2 |
| *Pseudomonas_E* | 0.0 | 0.0 | 0.7 | 1.1 | 0.1 | 0.1 | 0.0 | 0.0 | 0.1 | 0.0 | 29.0 | 31.9 | 0.0 | 0.0 | NI | NI | NI | NI | NI | NI |
| *Thiovibrio* | 0.0 | 0.0 | NI | NI | 0.1 | 0.1 | 0.5 | 0.6 | 1.7 | 2.6 | 2.8 | 6.9 | 0.3 | 0.3 | 0.1 | 0.0 | NI | NI | NI | NI |
| *UBA10092* | 2.0 | 1.5 | 3.1 | 0.2 | 0.0 | 0.1 | 0.0 | 0.0 | 0.0 | 0.0 | 0.8 | 0.8 | 1.8 | 1.3 | 2.7 | 0.6 | 2.5 | 0.5 | 1.6 | 1.8 |
| *UBA10183* | 4.1 | 2.9 | 4.2 | 0.3 | 0.0 | 0.0 | 0.0 | 0.0 | 0.0 | 0.0 | 0.3 | 0.4 | 1.0 | 0.9 | 2.8 | 1.3 | 2.6 | 0.6 | 2.7 | 0.7 |
| *UBA1562* | 2.6 | 1.8 | 3.5 | 0.0 | 0.0 | 0.0 | 0.0 | 0.0 | 0.0 | 0.0 | 0.5 | 0.6 | 0.9 | 1.0 | 2.3 | 1.2 | 2.1 | 0.4 | 2.0 | 1.1 |
| *JAAGZL01* | NI | NI | NI | NI | 0.0 | 0.0 | 0.0 | 0.0 | 0.0 | 0.0 | 0.4 | 1.0 | 3.1 | 3.4 | 2.9 | 4.2 | 0.3 | 0.1 | 0.0 | 0.0 |
| *Nocardioides* | NI | NI | NI | NI | 3.5 | 3.0 | 0.2 | 0.0 | 0.2 | 0.0 | 0.4 | 0.6 | 0.0 | 0.0 | NI | NI | NI | NI | NI | NI |
| *Sphingomicrobium* | NI | NI | NI | NI | 2.7 | 1.5 | 5.6 | 0.7 | 0.7 | 0.3 | 0.1 | 0.1 | 0.0 | 0.0 | NI | NI | NI | NI | NI | NI |

NI, not identified

**Supplemental Table 6** 16S rRNA gene based pairwise comparisons of ASVs using MANOVAs on a Bray Curtis dissimilarity matrix with 9999 permutations with only the biofilm stainless steel (SS) and Teflon (Tef) container results displayed. The replicate numbers for the different samples are given in the table. Significance defined as *p*<0.05 (all non-significant) with FDR adjustment.

|  | 1.B_SS (*n*=6) | 2.B_SS (*n*=2) | 4.B_SS (*n*=3) | 1.B_Tef (*n*=5) | 2.B_Tef (*n*=3) |
| --- | --- | --- | --- | --- | --- |
| 2.B_SS (*n*=2) | 0.134 | - | - | - | - |
| 4.B_SS (*n*=3) | 0.131 | 0.204 | - | - | - |
| 1.B_Tef (*n*=5) | 0.407 | 0.204 | 0.134 | - | - |
| 2.B_Tef (*n*=3) | 0.111 | 0.204 | 0.204 | 0.131 | - |
| 4.B_Tef (*n*=2) | 0.132 | 0.407 | 0.204 | 0.16 | 0.204 |

Abbreviations: The codes are the incubation time of 0, 1, 2, and 4 years plus biofilm (B) for the stainless steel (SS) and Teflon (Tef) containers.

**Supplemental Table 7** Statistical support for comparison of the 16S rRNA gene-based beta diversity based upon ANOVA and PERMANOVA plus comparison of 16S rRNA gene ASVs at the family level based upon Pairwise Wilcoxon significance comparison and effect testing between incubation years of the populations for the biofilms for SS and Tef one- and four-year incubations.

ANOVA dispersion

Response: Distances

Df SumOfSqs R2 F Pr(>F)

Model 1 0.041847 0.041847 2.0813 0.1711

Residual 14 0.281480 0.020106

PERMANOVA:

Permutation test for adonis under reduced model

Permutation: free

Number of permutations: 9999

adonis2(formula = biofilm. bray curtis dissimilarity ~ Incubation time in yrs * container types, data = samples_nmds_perm)

Df SumOfSqs R2 F Pr(>F)

Model 3 1.7408 0.36387 2.288 0.0014**

Residual 12 3.0434 0.63613

Total 15 4.7842 1.00000

---

Signif. codes: 0 ‘***’ 0.001 ‘**’ 0.01 ‘*’ 0.05 ‘.’ 0.1 ‘ ’ 1

**Pairwise Wilcoxon significance comparisons and effect of individual biofilm populations for one- and four-years incubation for SS and Tef samples.** Positive effect means the relative abundance for the family increased from one- to four-year incubation time with negative values meaning a decrease in relative abundance. Only families with a statistically significant increase or decrease (*p*<0.05) are included.

| **Family** | ***p* adj value** | **Wilcoxon**  **Year 1** | **Wilcoxon**  **Year 4** | **Wilcoxon effect** |
| --- | --- | --- | --- | --- |
| Anaerolineaceae | 0.000458 | 0.00808 | 0.0414 | 0.0333 |
| Nocardioidaceae | 0.000458 | 0.0253 | 0.00247 | -0.0229 |
| Other | 0.000458 | 0.415 | 0.73 | 0.315 |
| UBA5619 | 0.000458 | 0.00231 | 0.044 | 0.0417 |
| Villigracilaceae | 0.000458 | 0.000797 | 0.0238 | 0.023 |
| Acidaminobacteraceae | 0.00183 | 0.0249 | 0.00171 | -0.0232 |
| Desulfitobacteriaceae | 0.002 | 0.0583 | 0.000278 | -0.058 |
| Clostridiaceae | 0.00321 | 0.0342 | 0.0058 | -0.0284 |
| Sphingomonadaceae | 0.00549 | 0.0315 | 0.0118 | -0.0196 |
| LZORAL124-64-63 | 0.00622 | 0.00135 | 0.00663 | 0.00528 |
| Prolixibacteraceae | 0.0133 | 0.0515 | 0.00216 | -0.0493 |
| Holophagaceae | 0.014 | 0.0138 | 0.000637 | -0.0131 |
| UBA9217 | 0.0317 | 0.000193 | 0.00191 | 0.00172 |
| Acidobacteriaceae | 0.0364 | 0.0149 | 0.000207 | -0.0147 |

**Supplemental Table 8** Relative abundance and pairwise Wilcoxon comparisons and effect of 16S rRNA gene-based taxa with predicted streamlined genomes in the biofilm and outlet communities. Adjusted *p* values and effect are given for the comparison of the respective phyla between one- and four-years of incubation.

| **Phyla** | **Sample** | **Year** | **Relative abundance (%)** | ***p adj* value** | **Effect** |
| --- | --- | --- | --- | --- | --- |
| Aenigmatarchaeota | Biofilm | 1 | 0.0000 | NA | NA |
|  |  | 4 | 0.0020 |  |  |
|  | Outlet | 1 | 0.0821 | 0.622 | 0.0019 |
|  |  | 4 | 0.2630 |  |  |
| B1Sed10-29 | Biofilm | 1 | 0.0000 | NA | NA |
|  |  | 4 | 0.0017 |  |  |
|  | Outlet | 1 | 0.0000 | NA | NA |
|  |  | 4 | 0.0017 |  |  |
| Hadarchaeota | Biofilm | 1 | 0.0000 | NA | NA |
|  |  | 4 | 0.0000 |  |  |
|  | Outlet | 1 | 0.0011 | 0.778 | <0.0001 |
|  |  | 4 | 0.0109 |  |  |
| Hydrothermarchaeota | Biofilm | 1 | 0.0000 | NA | NA |
|  |  | 4 | 0.0059 |  |  |
|  | Outlet | 1 | 0.0071 | 0.778 | -0.0002 |
|  |  | 4 | 0.0067 |  |  |
| Iainarchaeota | Biofilm | 1 | 0.0000 | NA | NA |
|  |  | 4 | 0.0001 |  |  |
|  | Outlet | 1 | 0.0229 | 0.778 | 0.0005 |
|  |  | 4 | 0.0890 |  |  |
| Micrarchaeota | Biofilm | 1 | 0.0000 | NA | NA |
|  |  | 4 | 0.0111 |  |  |
|  | Outlet | 1 | 0.0147 | 1 | <-0.0001 |
|  |  | 4 | 0.0243 |  |  |
| Patescibacteriota | Biofilm | 1 | 1.16 | 0.00183 | 0.0398 |
|  |  | 4 | 4.61 |  |  |
|  | Outlet | 1 | 24.5 | 0.778 | -0.0408 |
|  |  | 4 | 20.3 |  |  |
| Undinarchaeota | Biofilm | 1 | 0.0000 | NA | NA |
|  |  | 4 | 0.0000 |  |  |
|  | Outlet | 1 | 0.0029 | 0.778 | 0.0001 |
|  |  | 4 | 0.0206 |  |  |

**Supplemental Fig. 1** The four containers in the Äspö HRL tunnel (a), connected to borehole HA0760B via a four-way splitter (b), and filled with Äspö diorite macadam (c).


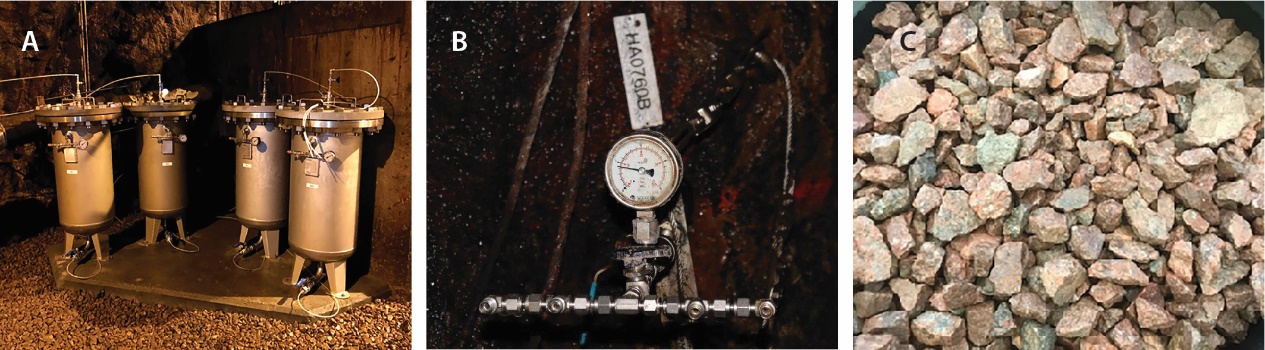


**Supplemental Fig. 2** Flow chart of the samples taken from the groundwaters, biofilm on the solid support, and planktonic cells flowing out of the four containers. The samples taken were (i) *in situ* borehole “groundwater” (in blue) from the four-way splitter attached to the valve directly from the borehole outlet (see Supplemental Fig. 1b) prior to the start of the experiment on 1 December 2020 and after the end of the experiment on 25 April 2025; (ii) “macadam” (in grey; see Supplemental Fig. 1c) sampled *ex situ* before it was used to fill the containers at the start of the experiment on 10 December 2020 and each time the containers were emptied for sampling the biofilm and then refilled with fresh macadam on 14 December 2021 and 6 December 2022; (iii) “outlet” waters (in green) of groundwater after it has passed through the containers at the start of the initial set-up on 10 December 2020 and after they had been emptied for sampling and then started again with fresh macadam on 20 December 2021 and 23 February 2023; (iv) “outlet” waters (in brown) of groundwater after it has passed through the containers before they were emptied on 6 December 2021, 6 December 2022, and 10 January 2025 plus on 15 December 2023 when the containers were not opened; (v) “biofilm” (in red) from various depths within the containers captured after one-, two-, and four-years incubation from the macadam surface after the containers had been opened and emptied on 14 December 2021, 6 December 2022, and 23 January 2025; and (vi) “blank” contamination control filters (in black) were frozen in liquid nitrogen and treated as per the sample filters on the nine indicated occasions. Number of replicates (*n*) is given for each sample.


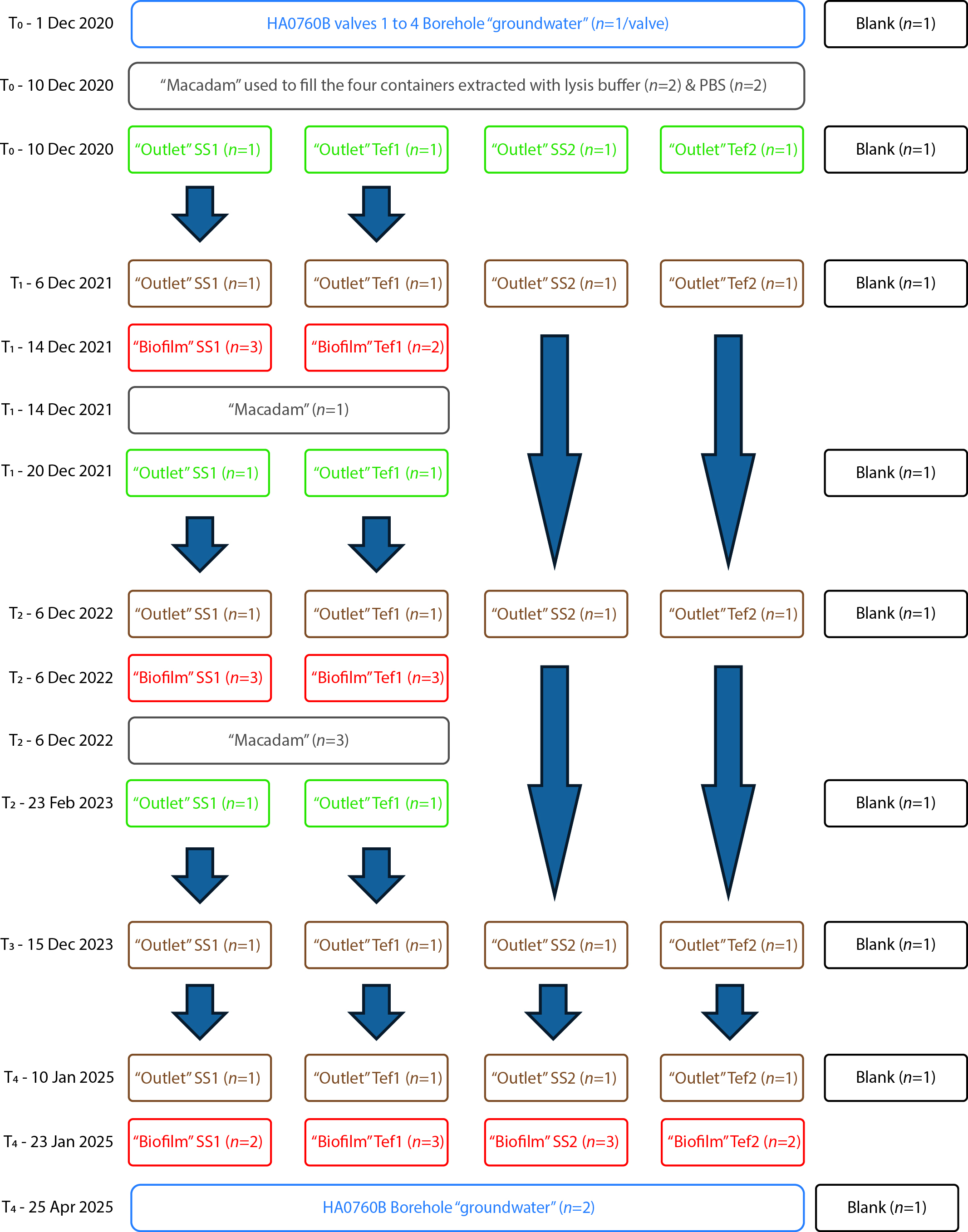


**Supplemental Fig. 3** Rarefaction curves of groundwater, biofilm, and planktonic samples showing ASV count versus read numbers.


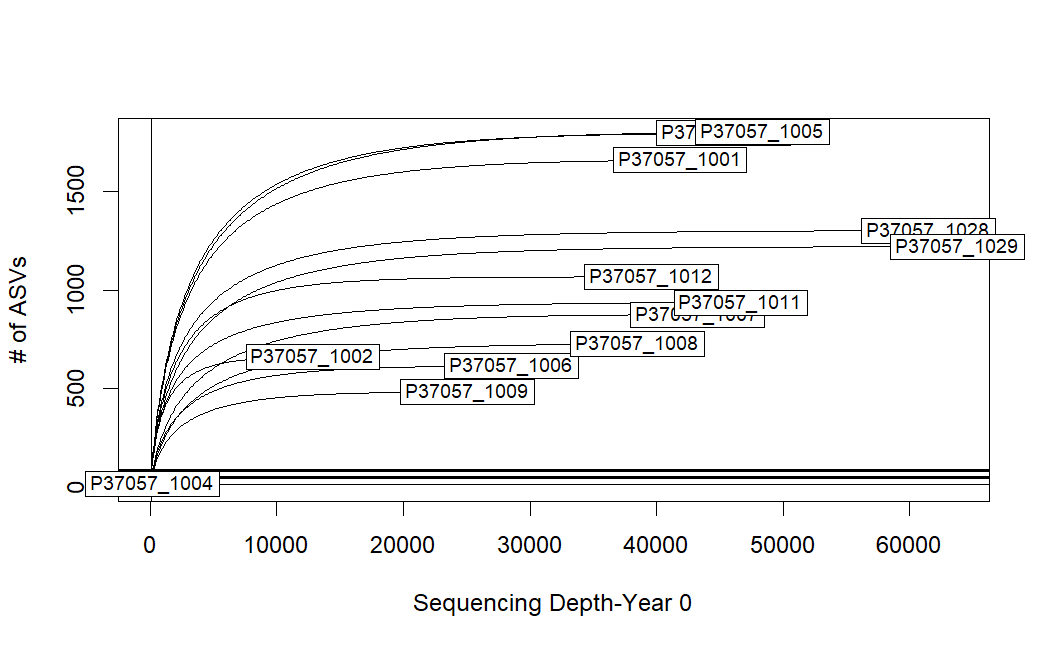


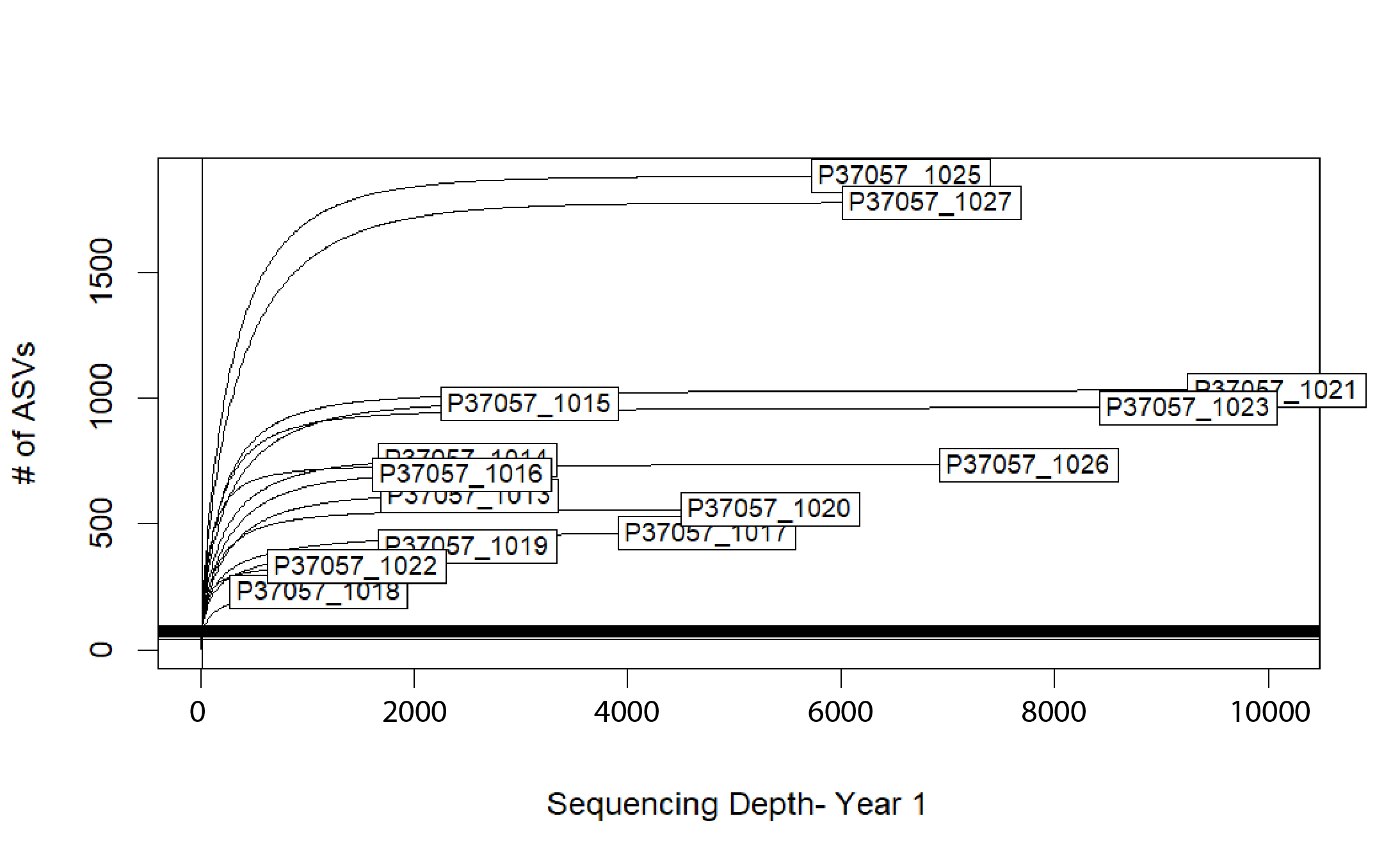


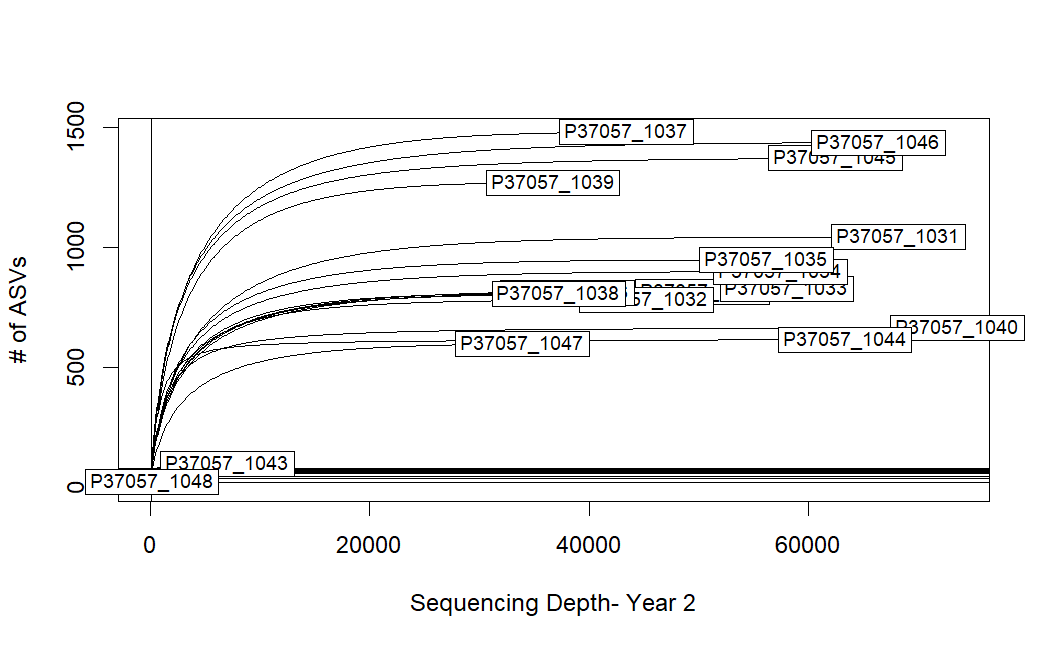


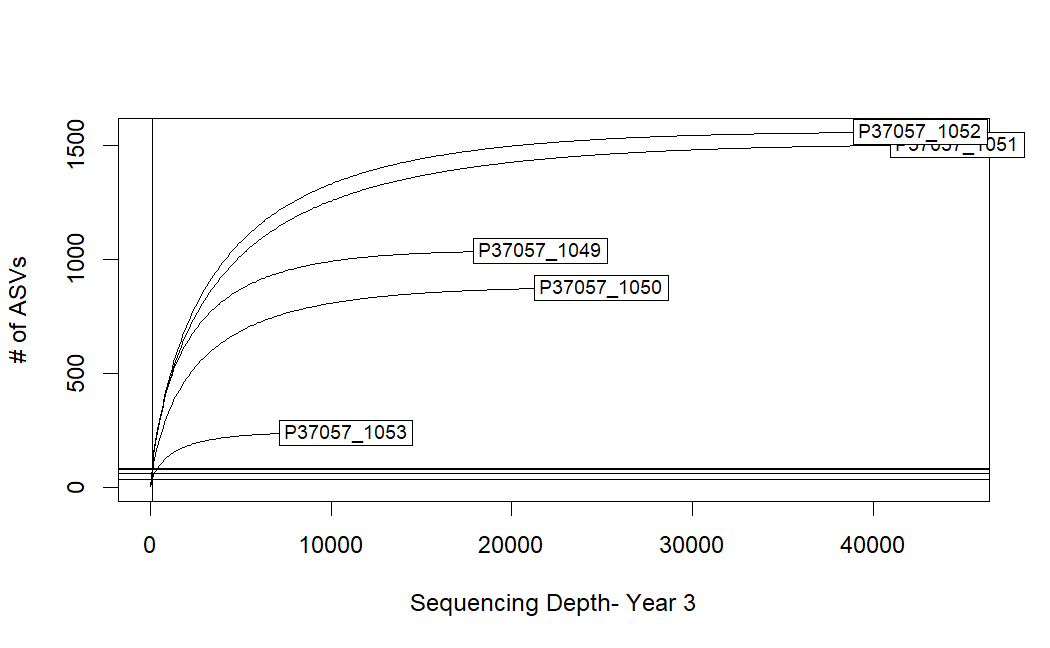


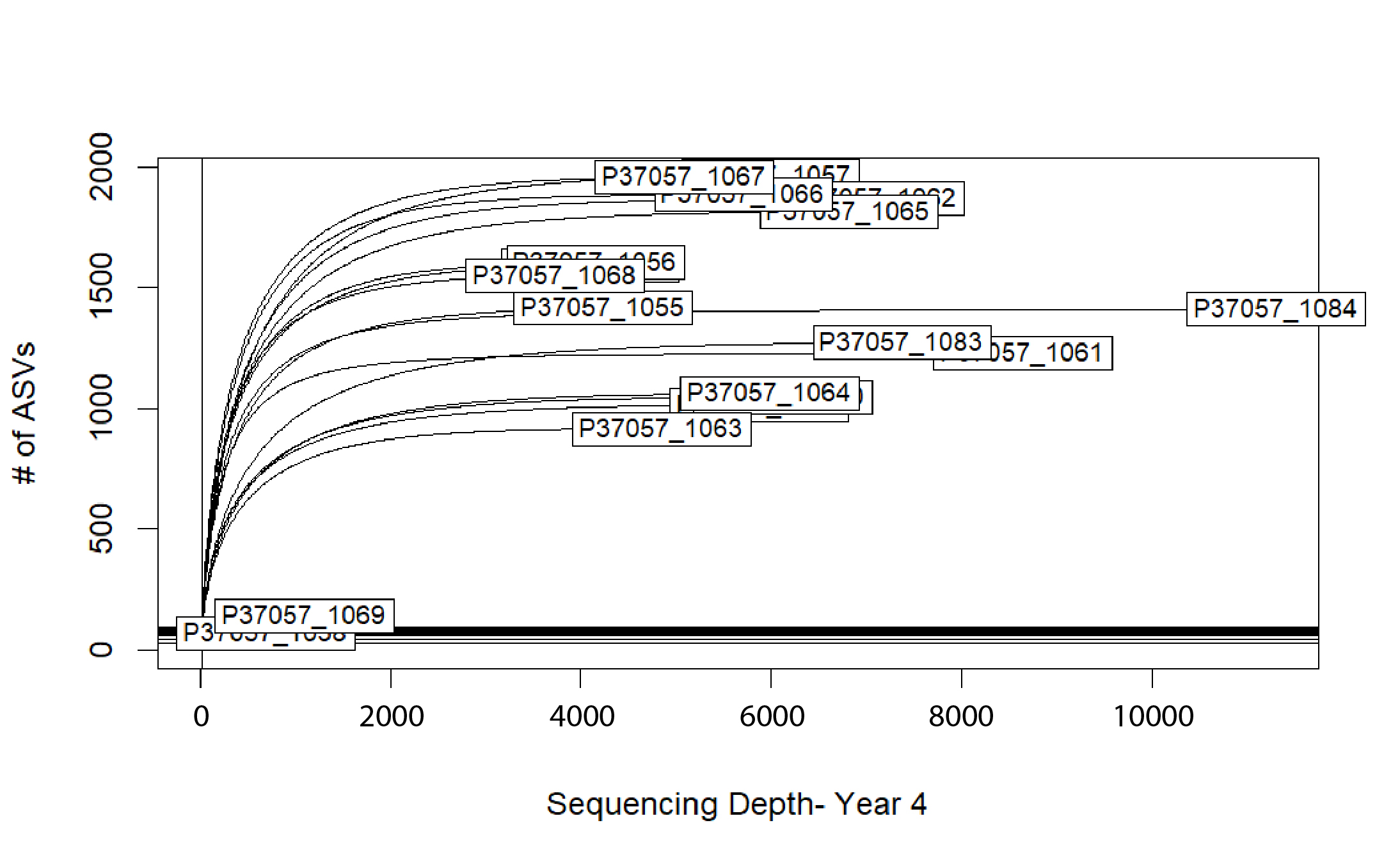


**Supplemental Fig. 4** 16S rRNA gene-based groundwater ASV stacked bar graphs for the individual groundwater samples taken prior and after the containers were attached to the HA0760B borehole, planktonic samples flowing out of the containers, and biofilm samples from the four containers. The figure is organized based upon sampling time in years and the top 20 families are given with the remaining sequenced grouped into “other”. Sampling points T_0_ and T_1_ are below with years T_2_ to T_4_ on the next page.


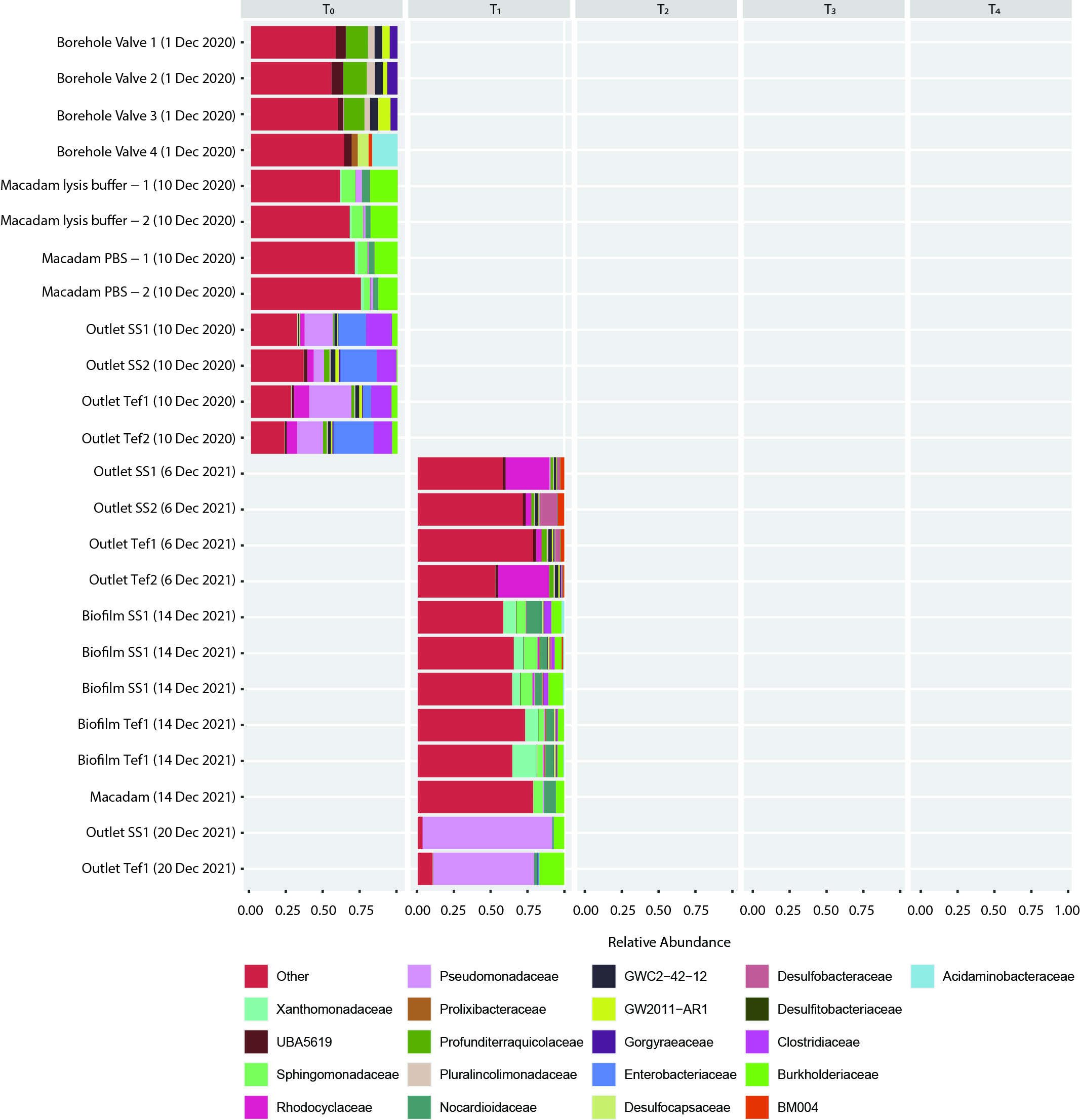


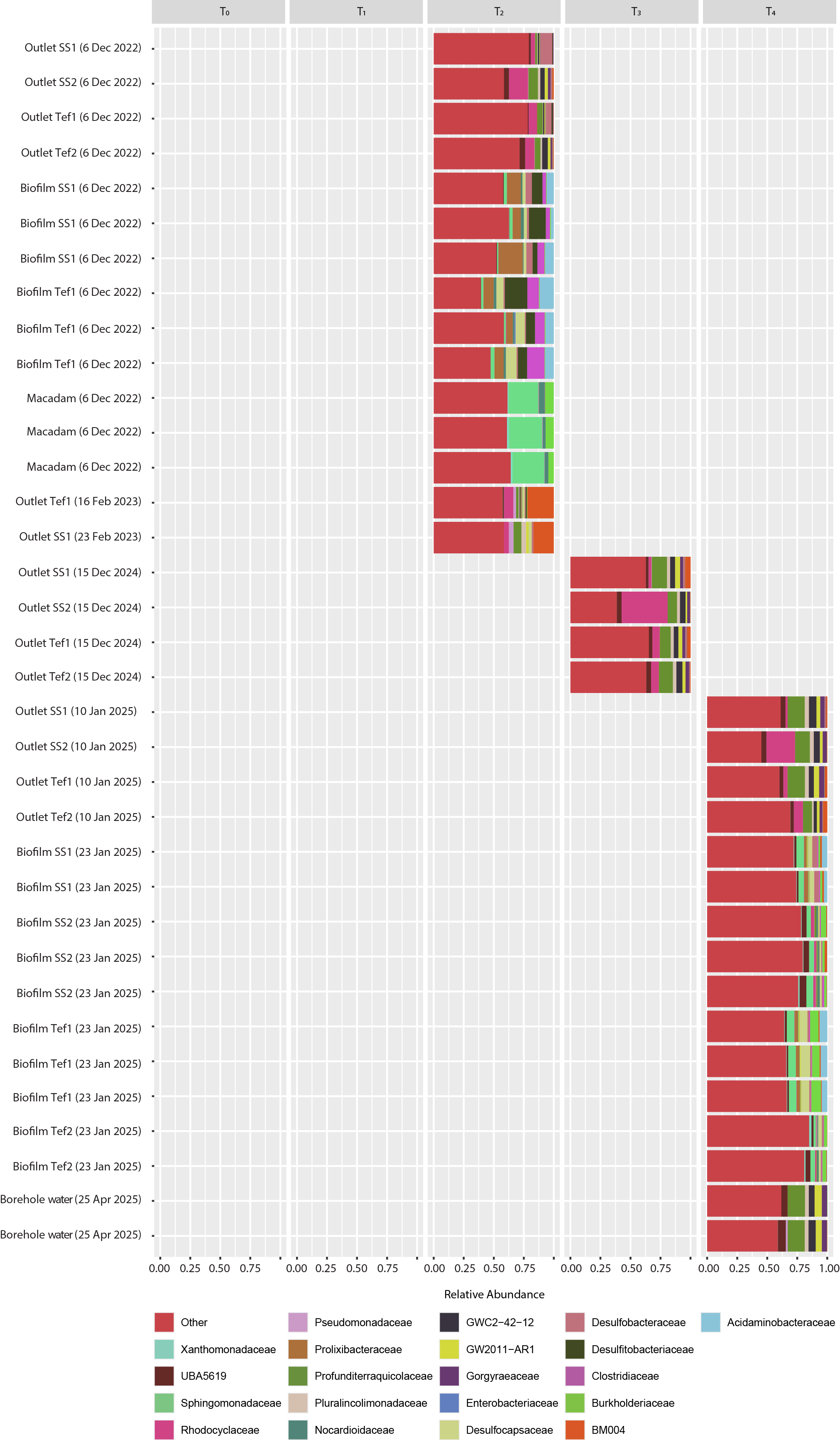


**Supplemental Fig. 5** Stacked bar graphs of 16S rRNA gene-based microbial communities from the borehole groundwater, biofilm, and the outlet water from the stainless steel and Teflon lined containers. The figure is organized based upon the incubation time in years according to the study flow diagram in Supplemental Fig. 2 and the top 20 taxa are given with the remaining sequenced grouped into “other”.

Class


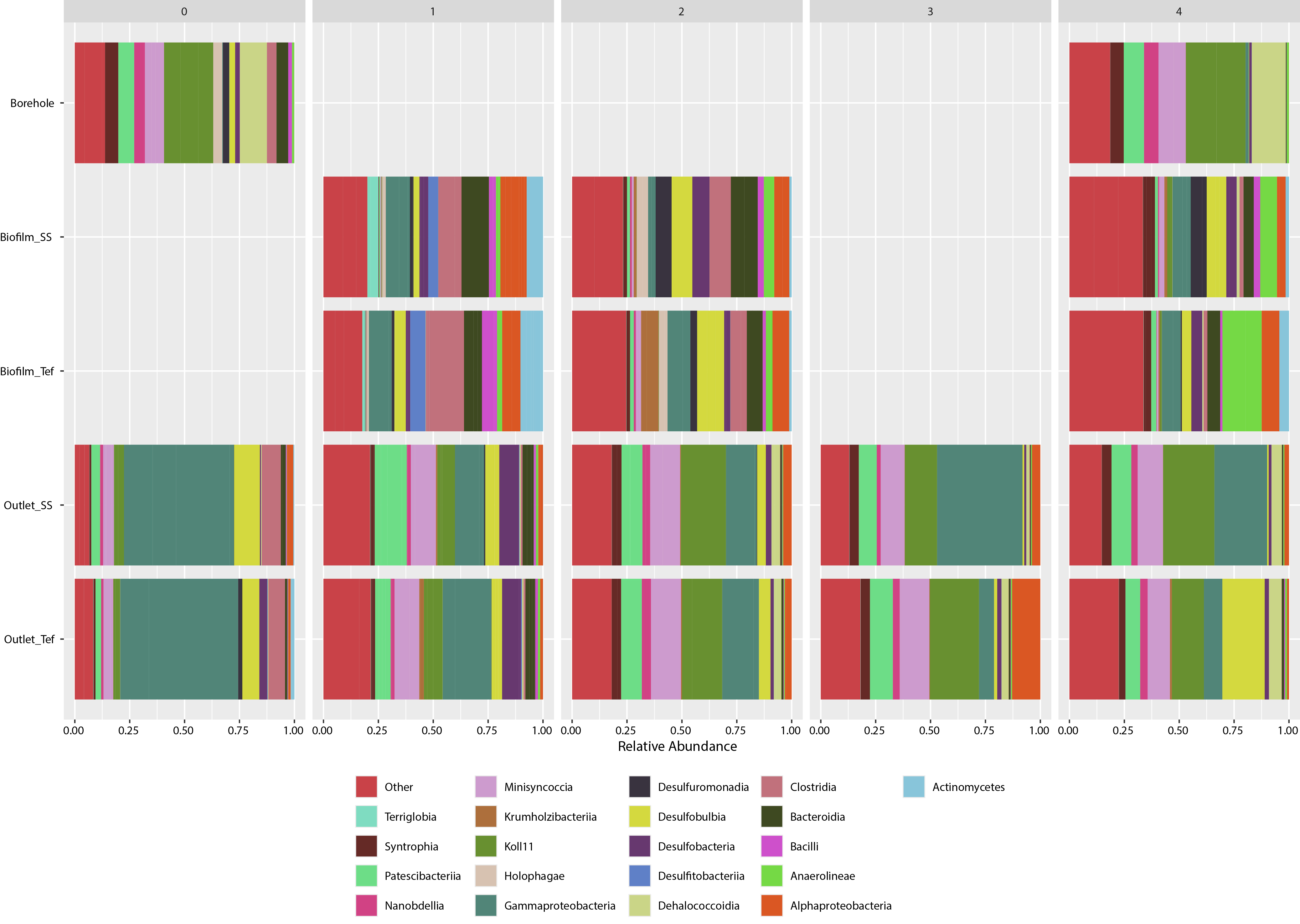


Order


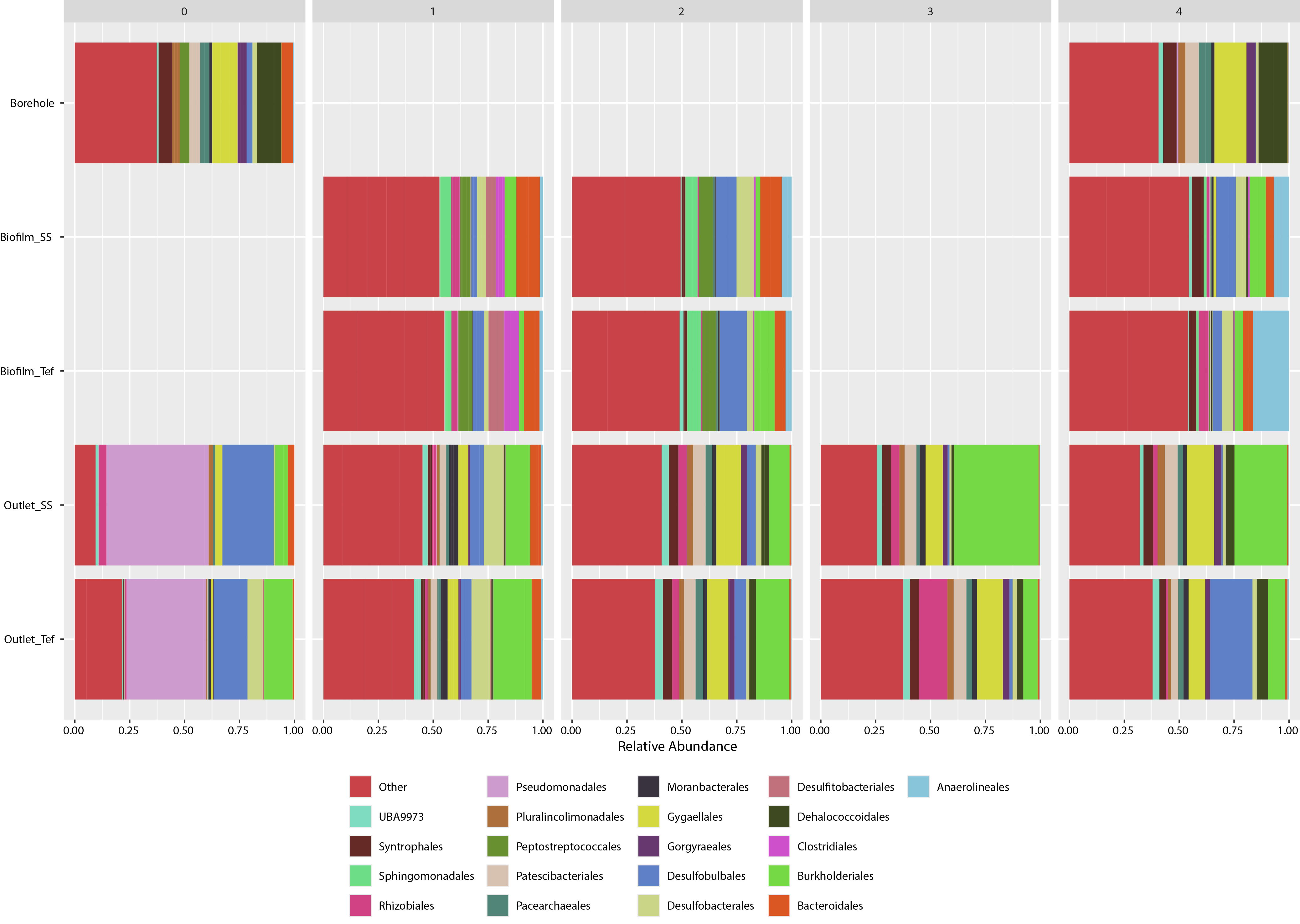


Genus


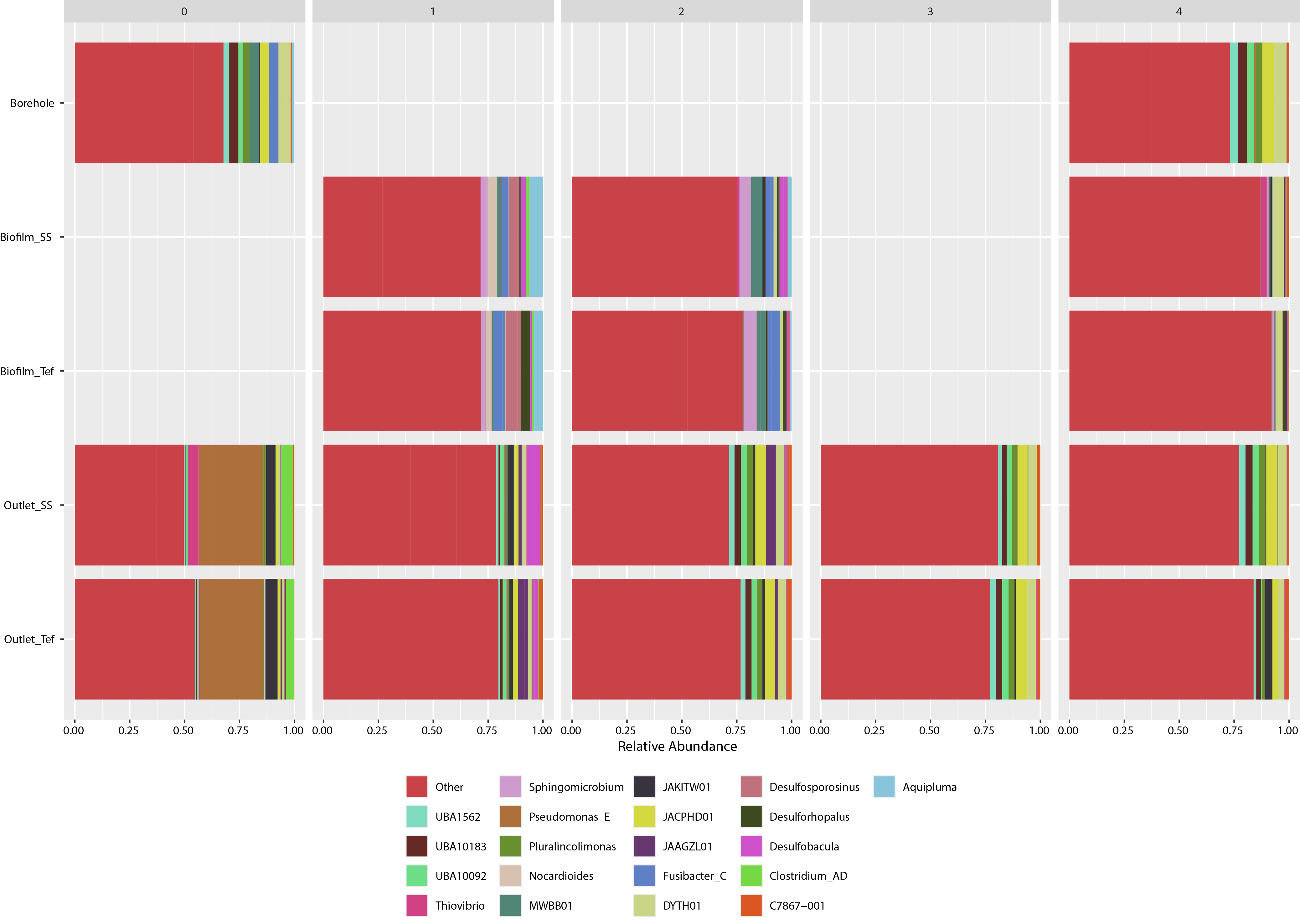


**Supplemental Fig. 6** Stacked bar graphs of 16S rRNA gene-based families from the biofilm on the macadam surface within the stainless steel and Teflon lined containers. The figure is organized based upon the incubation time in years according to the study flow diagram in Supplemental Fig. 2 and the top 20 families are given with the remaining sequenced grouped into “other”.


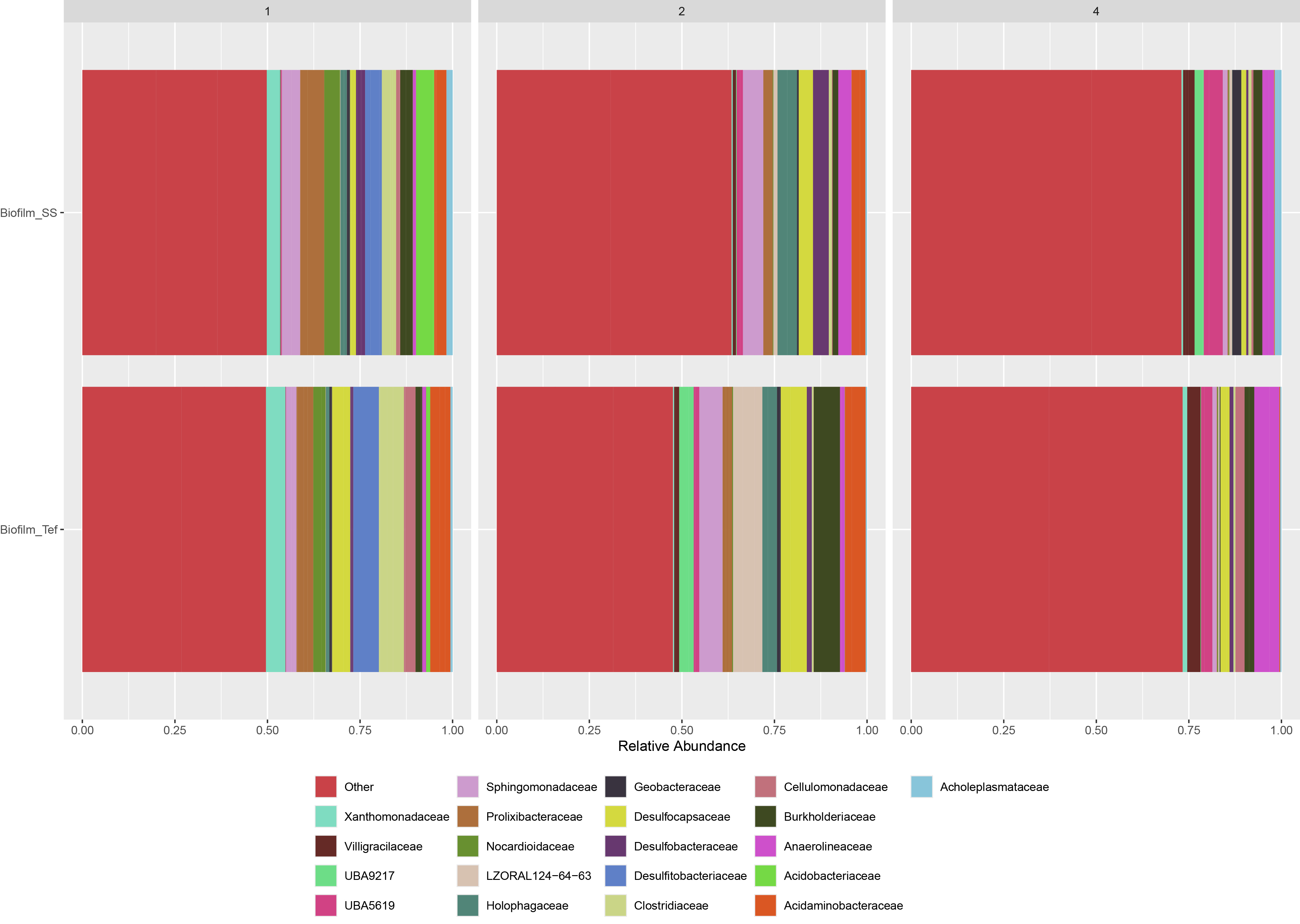


**Supplemental Fig. 7** Complete PiCRUSt2 analysis based upon the gene list in Supplemental Table 4. The categories refer to: A. hydrogen oxidation; B. sulfur oxidation; C. methane oxidation (methanotrophy); D. dissimilatory nitrate reduction; E. dissimilatory sulfate reduction; F. arsenate plus selenate reduction; G. methanogenesis; H. carbon fixation; and I. nitrogen fixation.


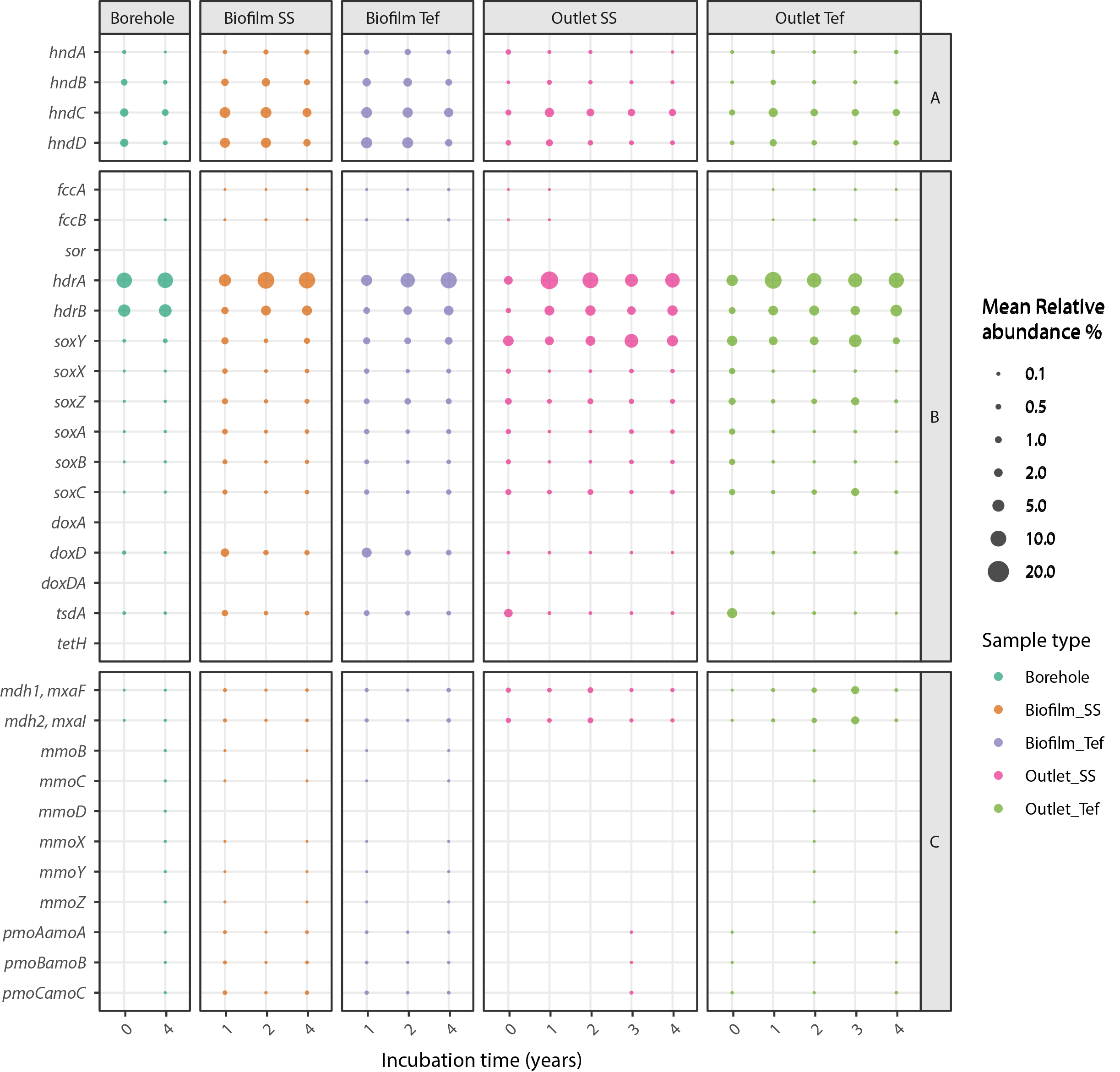


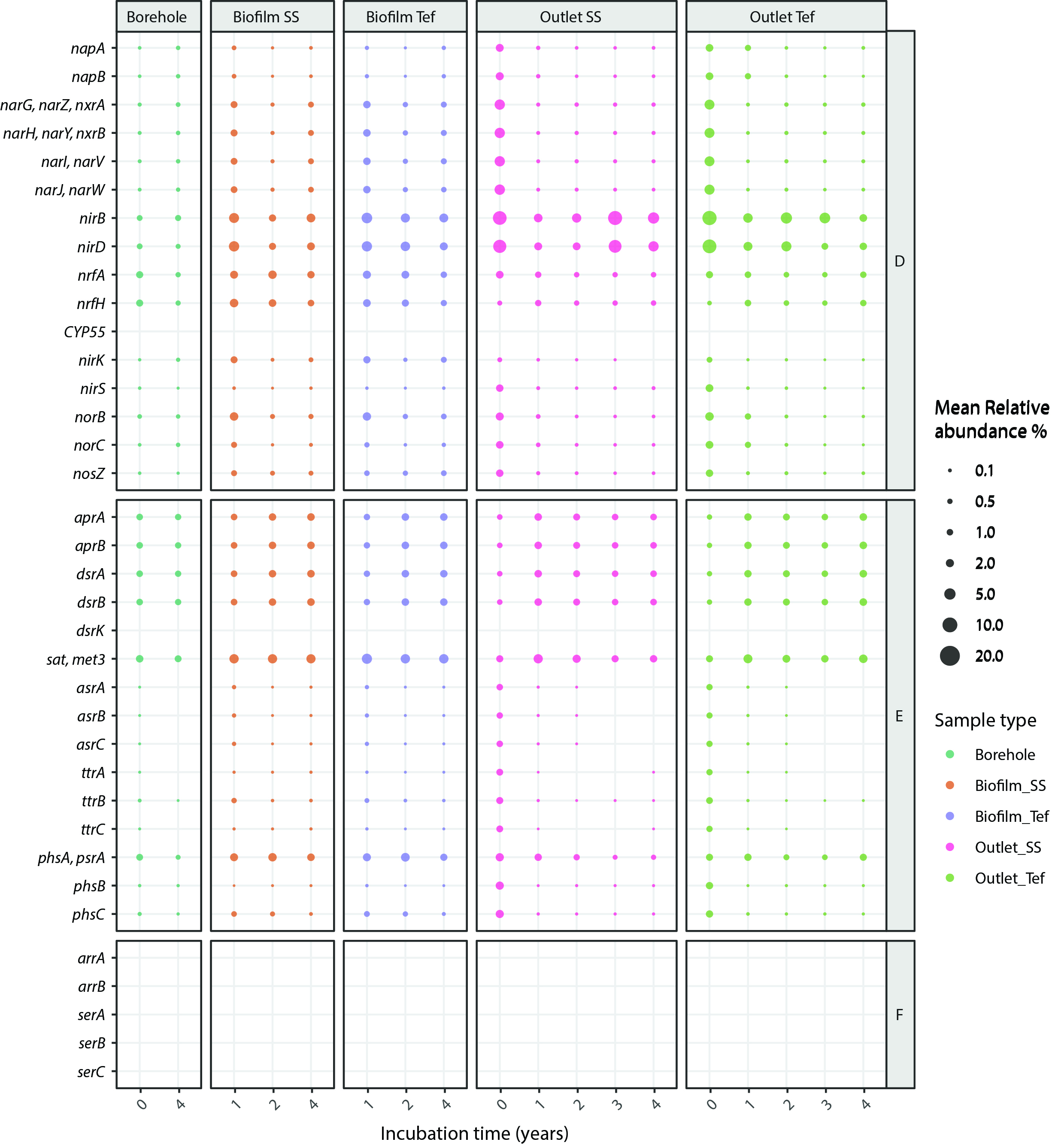

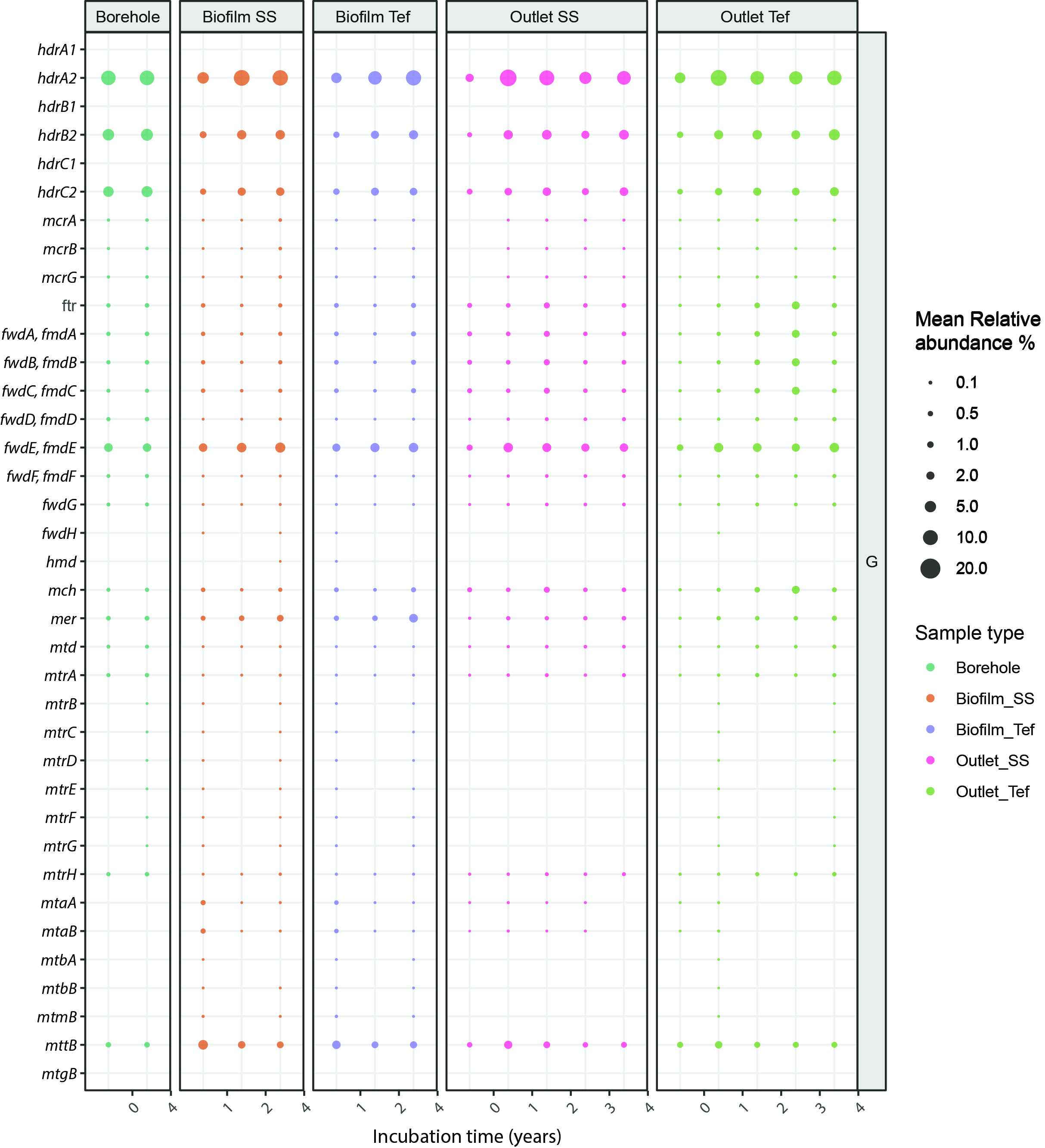

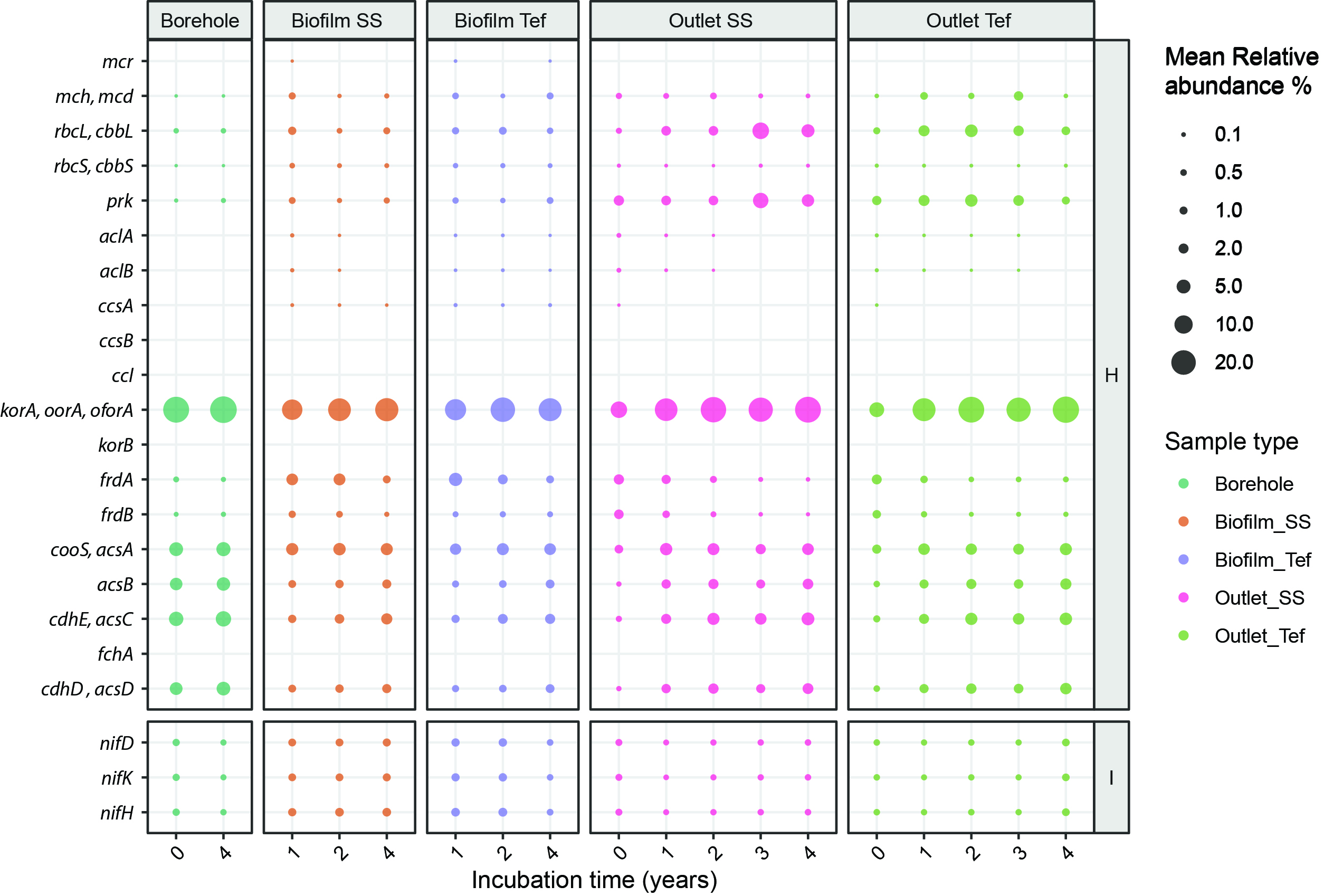

Supplement: Supplementary file 1 — Supplementary file1 (DOCX 16757 KB) [file 248_2026_2812_MOESM1_ESM.docx]
